# Genomic and immunogenic changes of *Piscine novirhabdovirus* (Viral Hemorrhagic Septicemia Virus) over its evolutionary history in the Laurentian Great Lakes

**DOI:** 10.1371/journal.pone.0232923

**Published:** 2021-05-28

**Authors:** Megan D. Niner, Carol A. Stepien, Bartolomeo Gorgoglione, Douglas W. Leaman

**Affiliations:** 1 Department of Environmental Sciences, University of Toledo, Toledo, Ohio, United States of America; 2 School of Oceanography, University of Washington, Seattle, WA, United States of America; 3 Genetics and Genomics Group, NOAA Pacific Marine Environmental Laboratory, Seattle, Washington, United States of America; 4 Department of Biological Sciences, University of Toledo, Toledo, Ohio, United States of America; 5 Department of Biological Sciences, Wright State University, Dayton, Ohio, United States of America; Universidad Miguel Hernández de Elche, SPAIN

## Abstract

A unique and highly virulent subgenogroup (-IVb) of *Piscine novirhabdovirus*, also known as Viral Hemorrhagic Septicemia Virus (VHSV), suddenly appeared in the Laurentian Great Lakes, causing large mortality outbreaks in 2005 and 2006, and affecting >32 freshwater fish species. Periods of apparent dormancy have punctuated smaller and more geographically-restricted outbreaks in 2007, 2008, and 2017. In this study, we conduct the largest whole genome sequencing analysis of VHSV-IVb to date, evaluating its evolutionary changes from 48 isolates in relation to immunogenicity in cell culture. Our investigation compares genomic and genetic variation, selection, and rates of sequence changes in VHSV-IVb, in relation to other VHSV genogroups (VHSV-I, VHSV-II, VHSV-III, and VHSV-IVa) and with other Novirhabdoviruses. Results show that the VHSV-IVb isolates we sequenced contain 253 SNPs (2.3% of the total 11,158 nucleotides) across their entire genomes, with 85 (33.6%) of them being non-synonymous. The most substitutions occurred in the non-coding region (NCDS; 4.3%), followed by the *Nv-* (3.8%), and *M-* (2.8%) genes. Proportionally more *M*-gene substitutions encoded amino acid changes (52.9%), followed by the *Nv-* (50.0%), *G-* (48.6%), *N-* (35.7%) and *L-* (23.1%) genes. Among VHSV genogroups and subgenogroups, VHSV-IVa from the northeastern Pacific Ocean has shown the fastest substitution rate (2.01x10^-3^), followed by VHSV-IVb (6.64x10^-5^) and by the VHSV-I, -II and-III genogroups from Europe (4.09x10^-5^). A 2016 gizzard shad (*Dorosoma cepedianum*) from Lake Erie possessed the most divergent VHSV-IVb sequence. The *in vitro* immunogenicity analysis of that sample displayed reduced virulence (as did the other samples from 2016), in comparison to the original VHSV-IVb isolate (which had been traced back to 2003, as an origin date). The 2016 isolates that we tested induced milder impacts on fish host cell innate antiviral responses, suggesting altered phenotypic effects. In conclusion, our overall findings indicate that VHSV-IVb has undergone continued sequence change and a trend to lower virulence over its evolutionary history (2003 through present-day), which may facilitate its long-term persistence in fish host populations.

## Introduction

*Piscine novirhabdovirus*–a.k.a. Viral Hemorrhagic Septicemia Virus (VHSV)–causes one of the world’s most severe finfish diseases, infecting >140 farmed and wild fish species across the Northern Hemisphere [1, 2]. Like many other RNA viruses, VHSV possesses a short genome, lacks apparent proofreading, and has a rapid generation time [3–5]. These factors often lead to rapid evolution of rhabdoviruses, facilitating their adaptation to new hosts and environmental conditions [6–9].

**Fig 1:**
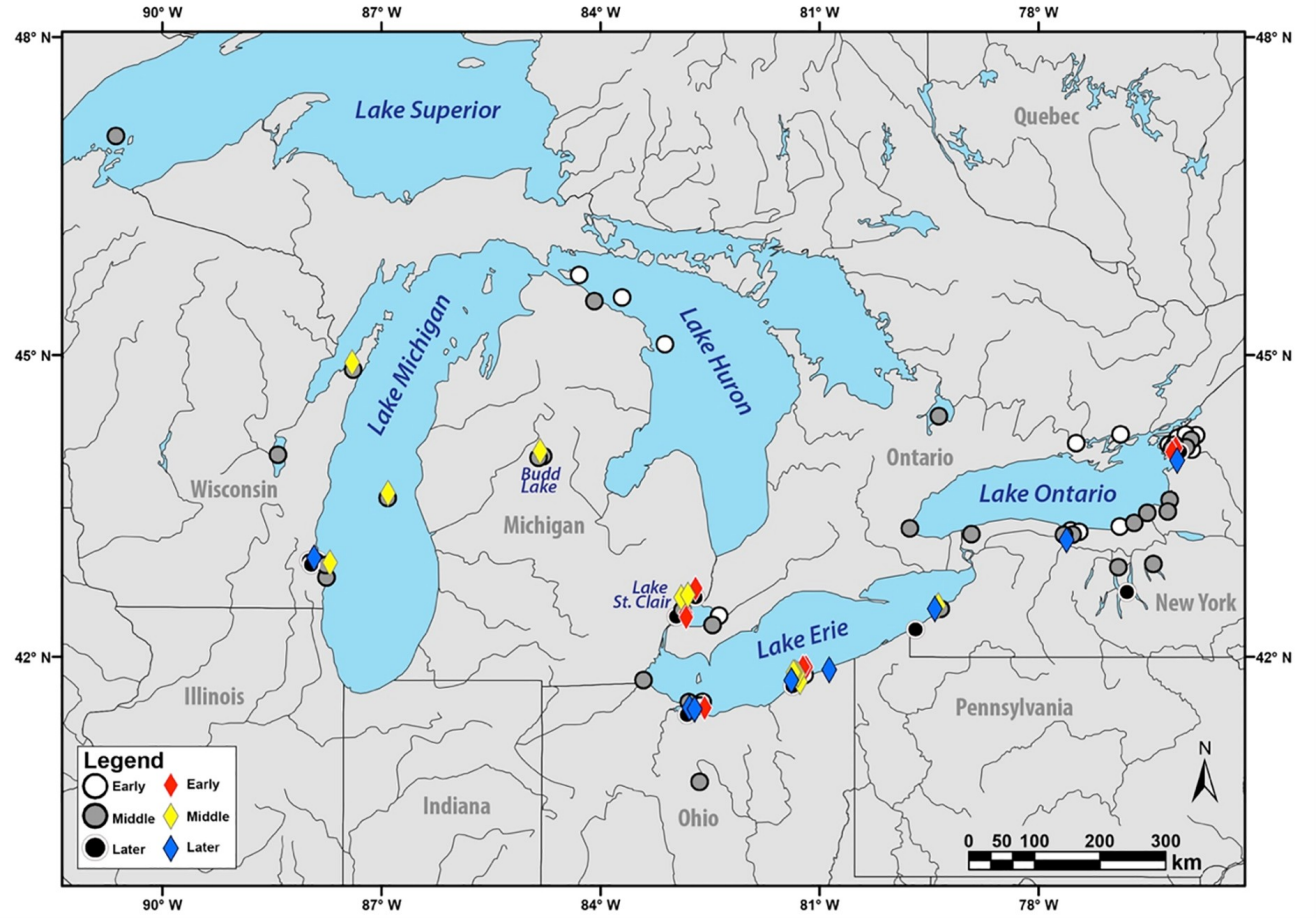
Map of VHSV-IVb occurrences in the Great Lakes region. Shapes are colored according to time periods of sample collections (Early: 2003–2006, Middle: 2007–2010, Later: 2011–2020). Diamonds denote isolates with sequenced whole genomes. Large circles depict VHSV-IVb isolates lacking whole genome data, at the time of this study. Map was created by MDN using ArcGIS software (https://www.esri.com) under the Department of Environmental Sciences of the University of Toledo’s license, and using freeware from Natural Earth (https://www.naturaalearthdata.com), a public domain site.

VHSV suddenly appeared in North America’s Laurentian Great Lakes in the mid-2000 decade, initially causing large outbreaks and mortality in 2005 and 2006, which thus far has affected >32 freshwater fish species [10–12]. This virus was designated as the geographically and genetically distinct subgenogroup VHSV-IVb, whose distribution and occurrences are mapped on Fig 1. Following its 2003–2006 emergence, VHSV-IVb outbreaks have become less prevalent [13–15], yet have been interspersed by smaller, geographically-restricted occurrences in 2007, 2009, 2011, and 2017 [13, 16]. Meanwhile, the VHSV-IVb virus has continued to diversify, according to sequence analyses of its selected gene regions [9, 14]. Its evolutionary trajectory appears to correspond to a “quasispecies” pattern of multi-directional, “cloud-like” diversification of closely related variants [7–9].

VHSV possess a single-stranded, negative sense RNA genome of 11,158 nucleotides (NTs), for which recombination has not been reported [17, 18]. It contains six genes: nucleoprotein (*N*), phosphoprotein (*P*), matrix (*M*), glycoprotein (*G*), nonvirion (*Nv*), and large protein (*L*) 5’*N*–*P*–*M*–*G*–*Nv*–*L*’3. VHSV belongs to the genus *Novirhabdovirus*, whose species also include *Salmonid novirhabdovirus* (IHNV), *Hirame novirhabdovirus* (HIRRV) and *Snakehead novirhabdovirus* (SHRV). All Novirhabdoviruses infect a wide variety of fish species and are united by their possession of a unique gene (*Nv*), which is located between the *G-* and *L*-genes [19]. The *Nv-*gene encodes a distinctive non-structural protein that is not found in the virions [2, 18, 19].

The functionality of this unique *Nv*-gene has been of scientific conjecture, which appears to vary among the four *Novirhabdovirus* species. Although non-essential for replication, *Nv* is required for pathogenicity in VHSV [19] and IHNV [20], but not in SHRV [21, 22]. Experiments that interchanged *Nv* between VHSV and IHNV discerned reduced pathogenicity in both reverse genetics systems [23]. *Nv* appears to function in suppressing host innate immune response [23–25] and in preventing apoptosis [19], inducing the viral-mediated host shutoff via the PERK-eIF2α pathway [26]. *Nv* also plays a strong immunogenic role by powerfully stimulating the type I interferon pathway in host cells [26, 27]. Single nucleotide polymorphism (SNP) changes in *Nv* can impact the capacity to suppress host cell responses [28, 29]. Although the presence of *Nv* characterizes Novirhabdoviruses, its sequence is not conserved among species [18].

VHSV comprises four described, geographically and genetically distinct genogroups: VHSV-I, -II, and -III originally occurred in Europe, and VHSV-IV in North America and Asia [30, 31]. VHSV-I is the original and oldest-discerned genogroup (dating to the 1930s), which possesses the largest known geographic range, the most named phylogenetic subgroups, and the greatest number of described host species [9]. Phylogenetic analyses have discerned that VHSV-I, -II, and -III shared a common evolutionary ancestry–separate from genogroup VHSV-IV, which is the sister group to the other genogroups [9, 14, 15]. VHSV-IV comprises three designated subgenogroups, with VHSV-IVa initially described from the northeastern Pacific coastal region in the 1980s [32–34]. In the late 1990s, VHSV-IVa was discovered off Korea and Japan (along the northwestern Pacific coast) [35]. Subgenogroup VHSV-IVc occurs in northwestern Atlantic marine waters, where it was discovered in 2000 [36]. Phylogenetic analyses place VHSV-IVc as the sister subgenogroup to VHSV-IVb, which clade (VHS-IVb + VHSV-IVc) then comprises the sister group to VHSV -IVa [9, 14, 15]. A fourth putative subgenogroup, VHSV-IVd, appears derived from VHSV-IVa and was isolated from wild lumpfish (*Cyclopterus lumpus*) in Iceland [37]; it remains to be fully described and sequenced.

VHSV pathology studies largely have used the original VHSV-IVb 2003 isolate (M103GL; here named C03MU; GenBank: GQ385941) [38–40] or tested experimentally mutated versions [19, 41, 42], leaving uncertainty about the traits of new variants. Just two other investigations (that we know of),have compared adaptive traits of the original VHSV-IVb 2003 isolate to those of other naturally occurring VHSV-IVb isolates. Notably, Imanse et al. [43] found that an isolate from a 2010 round goby (*Neogobius melanostomus*) in the St. Lawrence River, NY; GenBank: EF564588.1) showed reduced virulence and lower mortality in comparison to the original 2003 M103GL isolate (GenBank: GQ385941). A study by Getchell et al. [44] discerned lower viral titers, which did not lead to mortality, for a round goby isolate from 2006 and two gizzard shad isolates (from 2013 and 2014) in a laboratory challenge experiment that infected northern pike fry via immersion, versus greater virulence and mortality with the original 2003 VHSV-IVb isolate (M103GL = CO3MU).

Cellular recognition of viral byproducts triggers several host signaling cascades, including an array of Type I interferons (IFNs), which are highly induced by VHSV infection [45]. IFNs bind to specific surface receptors of effector cells, initiating transcription of IFN-stimulated genes to initiate antiviral response [46, 47]. Thus, comparatively measuring IFN production provides insights on how effectively the virus slows down the host’s response, serving as a proxy of potential host-pathogen evolution.

Host-pathogen relationships often are termed an evolutionary “arms-race”, described by Van Valen’s [48, 49] “Red Queen” hypothesis, in which selection pressures from pathogens lead either to death or adaptation in host populations, and *vice-versa*. Since RNA viruses mutate just below the maximum limit to maintain their functionality, they can readily adapt to the host’s defense modulations [50]. VHSV-IVb has been mutating over time since its appearance in the Great Lakes [11, 14, 15, 51], however, little is known about how its more recent isolates behave during infection. The rising importance of the aquaculture industry has raised concerns about fish pathogens, from economic and sustainability perspectives [52–54].

High-throughput sequencing (HTS) and contemporary bioinformatic techniques now allow analyses of entire viral genomes as a means of investigating evolutionary patterns in pathogen lineages [57]. Our study objective is to understand evolutionary relationships and trajectories across the VHSV-IVb genome in relation to host-pathogen coevolution. Only a few VHSV-IVb genomes were previously fully sequenced [28, 44, 54–56]: notably, just two other publications (that we know of) have evaluated their genomic similarities and differences, and those were limited to just a few isolates [44, 55]. In our study, we sequenced the entire genome of several recently retrieved isolates (from 2016), as well as a variety of older isolates to evaluate VHSV’s evolutionary patterns. Our present investigation compares these evolutionary patterns with those of other VHSV genogroups and with other Novirhabdoviruses. We further evaluate the differential pathogenicity and immunogenic effects of VHSV-IVb 2016 isolates on susceptible host cells, versus the original 2003 isolate. Additionally, we published a companion study of population genetic changes in VHSV-IVb over time, using these whole genome sequences in concert with a larger data set of partial *G*-gene sequences to analyze spatial and temporal patterns [51]. Knowledge of evolutionary changes across VHSV-IVb’s genome and the functional roles of its genes will aid our understanding of how such changes regulate the co-evolutionary “arms race” interactions between this pathogen and its hosts.

## Materials and methods

### Sampling and nomenclature

All fishes were collected by federal, provincial, and state agency collaborators, following their respective permits and regulations, and according to the University of Toledo Institutional Animal Care and Use Committee (IACUC) protocol #106419. VHSV-IVb samples from 2015–2016 were obtained, as described in Niner [57]. Archived isolates were provided by G. Kurath (USGS, Seattle, WA) as frozen infected media from Bluegill Fry (BF-2) cell culture (ATCC: CCL-91). Additional complete VHSV (I–IVa; S1 Table) and *Novirhabdovirus* (S2 Table) genomes were downloaded from NCBI GenBank and aligned using the program MEGA X [58]. Collection locations are provided in Fig 1. VHSV-IVb isolates are named here with unique identifiers, using the first letter of their lake name, the last two digits of the sampling year, followed by the first two letters of the host species’ common name. Example: the original isolate, collected from a Lake St. Clair muskellunge (*Esox masquinongy*) in 2003 here is termed C03MU (it also is known as M103GL; GenBank: GQ385941). If more than one isolate shared the above identifiers, an additional lower-case letter was added (S1 Table). We defined a haplotype as “a unique gene sequence differing by one or more nucleotide substitutions” from C03MU. Population genetic relationships, based on this dataset, are evaluated separately [51].

### Virus isolation

Frozen media was thawed on ice, and 30 μl from each sample was diluted 1:5 with serum-free Eagle’s Minimal Essential Medium (EMEM; Gibco), and added to individual wells of a 12 well plate with confluent BF-2 monolayers. Cells were incubated 1 h with the infected medium at 20°C, which then was replaced with complete EMEM 10% (v/v) cosmic calf serum (GE Healthcare) and 1% penicillin/ streptomycin antibiotics (Invitrogen). Infected cells were incubated at 20°C for ≤one week and sampled when ≥80% cytopathic effect (CPE) was achieved. Media was collected and added to a 1.5 ml tube with 250 μl of versene (Gibco) for 10 min, followed by 4 min 4,000 rpm centrifugation at 4°C. The supernatant was discarded, and the versene/cell mixture spun again, as before. The remaining supernatant again was discarded, and 250 μl Trizol® (Invitrogen) was added to the remaining pellet.

### Viral genome sequencing

cDNA was synthesized from total RNA extracted from the tissue samples using SuperScript IV (Invitrogen), following manufacturer’s instructions. Genomic cDNA was amplified in four segments using primers from Schönherz [59], substituting VHSV_Frag1I_nt18_+s with a more specific primer (5’GAGAGCTGCAGCACTTCACCG C3’), and 1 μl cDNA in 25 μl polymerase chain reactions (PCRs) with One Taq DNA polymerase (New England Biolabs). Amplicons were examined under UV light on 1% agarose gels stained with ethidium bromide. Target PCR products were gel-excised and purified using QIAquick Gel Extraction kits (Qiagen).

Genomic sequencing was outsourced to Ohio State University’s Molecular and Cellular Imaging Center (Wooster, OH). Sequences then were uploaded by us to the Galaxy web platform and analyzed with usegalaxy.org programs [60]. Segments were aligned to the reference VHSV-IVb genome (C03MU, GenBank: GQ385941) using MAP WITH BWA-MEM [61]. For each of the 48 isolates we sequenced here, consensus sequences were generated followed by manual checking of each single nucleotide polymorphism (SNP) and read coverage was determined using the Integrative Genomics Viewer (IGV) [62, 63].

Additional PCRs were conducted to amplify the front 700 nucleotides (NTs) and end 400 NTs of the genome, with 45 s extension time, in order to complete the whole genomes. The front segment utilized VHSV_Frag1I_nt18_+s (5’GAGTTATGTTACARGGGACAGG3’) [59] and anti-sense 5’TGACCGAGATGGCAGATC3’, and end primers were designed by us based on the VHSV-IVb original reference genome (GenBank: GQ385941) (End sense: 5’CCCAGATGCTATCACCGAGAA3’, End anti-sense: 5’ACAAAGAATCCGAGGCAGGAG3’). Those cleaned products were Sanger-sequenced at Cornell DNA Services (Ithaca, NY), and checked and aligned by us. The consensus sequences, front, and end segments then were concatenated, aligned, and trimmed using MEGA X [58], to complete the whole genome sequences.

### Genetic analyses

The Basic Local Alignment Search Tool (BLAST) was used to align the whole genome sequences. JMODELTEST v3.7 [64, 65] employed the Akaike Information Criterion (AIC) to determine the best-fit evolutionary models [65]. Phylogenetic analyses evaluated the most parsimonious evolutionary relationships (per [9, 14]) with Maximum Likelihood (ML) (PHYML v3.0) [66] and Bayesian (MRBAYES v3.1) [67] algorithms. For the latter, Metropolis-coupled Markov Chain Monte Carlo (MCMCMC) analyses were run for five million generations, sampling every 100 to obtain posterior probability (pp) values. Burn-in was determined by plotting the log likelihood values to identify when stationarity was reached, discarding the first 25%. Branch support for ML was calculated with non-parametric bootstrapping replications, using 500 for the VHSV analysis and 1450 for the VHSV-IV analysis. Haplotype networks were analyzed by us using PopART (Population Analysis using Reticulate Trees; http://popart.otago.ac.nz) with TCS [68].

### Evaluating evolution and selection

Comparative divergence times among VHSV taxa were estimated using BEAST v1.10.4 [69], using JMODELTEST output and a relaxed molecular clock with lognormal distribution, sampled every 50,000 of 500,000,000 generations. Outputs were assessed with TRACER v1.5 (in BEAST) to ensure stationarity. Collection dates were used as calibration points and the tree branches were set, following the PHYML output. Numbers of nucleotide substitutions per site per year (*k* = substitutions site–1 yr–1) were determined from the pairwise (p) distances. Nucleotide (NT) and amino acid (AA) substitutions were evaluated for all VHSV-IVb isolates and compared to the reference C03MU sequence (GenBank: GQ385941). Results from the VHSV-IVa isolates were compared to KRRV9601 (GenBank: AB179621), and VHSV-I+III with the European VHSV-Ia isolate Hededam (GenBank: Z93412).

Two codon-based methods examined the possibility of selection pressures for the entire genome and separately for each gene. Fast, unconstrained Bayesian approximations (FUBAR) [70] were used to identify positive or purifying selection, which are the pressures that select for beneficial traits. However, FUBAR’s assumption of constant selection may not accurately represent VHSV-IVb since selection pressures may differ across hosts and with environmental factors. To remedy this, we additionally used MEME (mixed effects model of evolution) [70, 71], which can detect positive selection under strong purifying selection or with removal of detrimental variants. FUBAR and MEME were run with HyPHY on DataMonkey (www.datamonkey.org), with their significance evaluated using the posterior probability of >0.95 for FUBAR and *p*<0.05 for MEME.

Amino acid sequences were submitted to the Phyre2 web portal (www.sbg.bio.ic.ac.uk/phyre2/) for protein modeling, prediction, and analyses [72]. For open reading frame (ORF) analysis, full length cDNA nucleotide sequences per isolate were submitted to the NCBI ORF Finder (www.ncbi.nlm.nih.gov/orffinder/) to search for alternate products >300 NTs in length. Any sequences that returned as coding in reverse were discounted because VHSV is single-stranded.

### Virulence and immunogenicity assessments

*Epithelioma papulosum cyprinid* (EPC) (ATCC: CRL-2872) and BF-2 cells were cultured, as described by Niner [57]. Three viable independent viral stocks from 2016 –isolates E16GSa (Cell16a) and E16LB (Cell16b and Cell16c)–were derived from pooled organs (kidney, liver, and spleen) of sampled fish. Cell culture amplified stocks are labelled “Cell” in our results. Due to low concentrations, the original E16LB sample was only partially sequenced, which matched E16GSa in those sequence regions [51, 57]. The cell culture amplified reference control (CellC03) was derived from C03MU. VHSV-IVb isolates were amplified for subsequent purification by infecting confluent monolayers of BF-2 cells in 15 cm tissue culture dishes with a 1:1000 (v/v) dilution of un-purified virus stock in serum-free EMEM. Viral adsorption occurred for 1 h, then the medium was replaced with complete EMEM. The plates were incubated at 20°C, until >75% CPE occurred at ~72 hours post infection (hpi). The virus containing media and the attached cells were collected and subjected to one freeze-thaw cycle, followed by a 30 min 4,000 rpm centrifugal removal of the debris at 4°C. The supernatant from each isolate was clarified using 0.22 μm syringe-tip filters, and the viral particles were purified through a 25% (w/v) sucrose pad upon 3 h of 25,000 rpm ultra-centrifugation at 4°C. Virus-containing pellets were re-suspended overnight at 4°C in phosphate buffered saline (PBS). Virus stocks were titered by 1:10 serial dilution using confluent EPC cells, divided into 100 μl aliquots, and stored at -80°C. Virus-induced CPE were quantified with a sulforhodamine B (SRB) assay [42].

Infected EPC cells were sampled respectively at 0, 18, 50, 72, or 96 hpi at a multiplicity of infection (MOI) = 1.0 for C03MU or Cell16-a, -b, or -c, and stored at -80°C. A viral yield assay compared the replication ability of the three test isolates of E16GSa to C03MU virus, by titering the media from each time point in 1:10 serial dilutions on BF-2 cells. Plaques were counted at 96 hpi, and the final viral concentration in plaque forming units per ml (pfu/ml) was calculated per each time point. Antiviral assays followed Ke et al. [42]. UV-irradiated media from each harvested time point was added to EPC cells in 1:3 serial dilutions for 24 h. Cells then were challenged with sucrose-purified VHSV-IVb C03MU for 96 h, fixed, and stained with crystal violet (Sigma-Aldrich). CPE plaques were counted and normalized to counts obtained from untreated and uninfected wells. One unit of IFN was defined as the dilution that conferred 50% protection from viral CPE, whose value then was used to measure the IFN activity (ml) in the testing culture media per time point.

Total RNA was extracted from the infected EPC cells, following a previously optimized TRIzol protocol [73]. One μg of each RNA sample was reverse transcribed into cDNA using a 10 min incubation at 70°C with 100 ng of random hexamer primer (Thermo Scientific) and water, for 7 μl total volume. Reactions were cooled to 4°C before adding 13 μl of Moloney Murine Leukemia Virus Reverse Transcriptase (M-MLV-RT) mixture [10X First Stand buffer, 10 mM dNTPs, 0.05 mM random hexamers, 25 U/μl RNasin Plus (Promega), and 200 U/μl M-MLV (Promega)], which then was incubated at 42°C for 1 h. cDNA was diluted 10-fold in water and archived at -80°C. 1 μl of each cDNA was tested using RT-qPCR, with 5 μl of the Radiant Green Lo-ROX 2X qPCR kit (Alkali Scientific), 50 ng of each oligonucleotide, and water to total 10 μl. Primers used were: VHSV-Nse/as, EPC IFN se/as, and β-actin se/as [43]. Reactions and data collections were performed on a C1000 Real Time Thermocycler (Bio-Rad), with initial 3 min denaturation at 95°C, followed by 40 cycles of 15 s at 95°C, and 30 s elongation at 60°C. Readings were recorded at the end of each elongation cycle, and the threshold values were obtained from an automated single point threshold within the log-linear range. Detections of VHSV-*N* and EPC IFN were normalized to EPC β-actin detection, and to the gene expression of uninfected cell samples. Relative gene expression used the 2^-ΔΔCT^ method [74].

## Results

### Genomic and genic changes

Our analyses of 44 VHSV-IVb whole genome sequences (11,083 NTs), plus an additional four from the literature (GenBank: GQ385941, KY359355–57), indicated no insertions or deletions (S1 Table). Of these, 39 had unique sequences (0.81), which differed by ≥1 NT from C03MU = GQ385941), and are deposited in GenBank (MK782981–MK783014). Isolates C06NP, C06RB, C06SR, C06YP, CO6FD, E06WBc, M08AMa,b, C08LEa,b, and C09MU were identical; these are designated here as the “C06NP group” (MK782990). No new ORFs were detected.

The 39 unique VHSV-IVb gene sequences contained a total of 253 SNPs (Table 1), with 85 (0.336) being nonsynonymous, encoding different AA. Most of the SNPs and the majority of AA changes occurred in the *L-*gene (112 SNPs, 38 AA) and the fewest in the *Nv-*gene (14 SNPs, 7 AAs), as anticipated from their respective lengths (*L*: 5955 NTs, *Nv*: 369). The NCDS, which does not encode amino acids, contained the highest proportion of SNPs (32 SNPs, 0.043 of the region), whereas the *P-* and *L-*genes had the lowest (0.019). The *Nv-*gene encoded the highest proportion of AA differences (0.057) and the *P-*gene had the least (0.014). The highest proportion of nonsynonymous: synonymous changes (dN/dS) was found in the *M-*gene (0.53) and the lowest in the *P-*gene (0.23). The mean dN/dS ratio was 0.166 for all VHSV-IVb genome sequences (see Table 1).

**Table 1:** Single nucleotide polymorphisms (SNPs) from each gene’s coding region and the combined non-coding regions (NCDS) for VHSV whole genome sequence variants of (A) VHSV-IVb, (B) VHSV-IVa, and (C) VHSV-I+III (combined).

**A. VHSV-IVb**							
**Region**	**Length**	**# changes**	**% changes**	**dN/dS**	**#Tv**	**Tv/Ts**	**Evolutionary Rate**
	**NTs (AAs)**	**NTs (AAs)**	**NTs (AAs)**				
***N*-gene**	1215 (405)	28 (10)	0.023 (0.025)	0.357	7	0.250	5.83E-05
***P*-gene**	669 (223)	13 (3)	0.019 (0.014)	0.231	2	0.154	7.18E-05
***M-*gene**	606 (202)	17 (9)	0.028 (0.045)	0.529	2	0.118	7.93E-05
***G*-gene**	1524 (508)	37 (18)	0.024 (0.035)	0.486	6	0.162	8.51E-05
***Nv*-gene**	369 (123)	14 (7)	0.038 (0.057)	0.500	4	0.286	9.76E-05
***L*-gene**	5955 (1985)	112 (38)	0.019 (0.019)	0.339	18	0.161	5.02E-05
**NCDS**	745 (N/A)	32 (N/A)	0.043 (N/A)	N/A	3	0.094	1.40E-04
**Total**	11083 (3446)	253 (85)	0.023 (0.025)	0.166	42	0.166	6.64E-05
**B. VHSV-Iva**							
***N*-gene**	**54 (17)**	**1215 (405)**	**0.044 (0.042)**	**0.315**	**6**	**0.111**	**3.14E-04**
***P*-gene**	45 (18)	669 (223)	0.067 (0.081)	0.400	10	0.222	1.22E-03
***M-*gene**	36 (14)	606 (202)	0.059 (0.069)	0.389	5	0.139	4.03E-04
***G*-gene**	87 (25)	1524 (508)	0.057 (0.049)	0.287	12	0.138	5.35E-04
***Nv*-gene**	31 (10)	369 (123)	0.084 (0.081)	0.323	3	0.097	1.07E-03
***L*-gene**	424 (119)	5955 (1985)	0.071 (0.059)	0.281	65	0.153	1.02E-03
**NCDS**	110 (N/A)	720 (N/A)	0.153 (N/A)	N/A	30	0.273	4.09E-03
**Total**	787 (203)	11058 (3446)	0.071 (0.059)	0.258	131	0.166	1.06E-03
**C. VHSV-I+III (combined)**							
***N*-gene**	**1215 (405)**	**149 (43)**	**0.123 (0.106)**	**0.289**	**34**	**0.228**	**4.59E-05**
***P*-gene**	669 (223)	73 (30)	0.019 (0.135)	0.411	13	0.178	4.07E-05
***M-*gene**	606 (202)	65 (15)	0.107 (0.074)	0.231	10	0.154	5.45E-05
***G*-gene**	1524 (508)	198 (40)	0.130 (0.079)	0.202	41	0.207	4.54E-05
***Nv*-gene**	369 (123)	76 (27)	0.206 (0.220)	0.355	14	0.184	7.78E-05
***L*-gene**	5955 (1985)	618 (70)	0.104 (0.035)	0.113	106	0.172	3.58E-05
**NCDS**	788 (N/A)	113 (N/A)	0.143 (N/A)	N/A	36	0.319	3.60E-05
**Total**	11126 (3446)	1292 (225)	0.116 (0.065)	0.174	254	0.197	4.09E-05

Numbers of nucleotides (NTs) are reported in front of the number of amino acid (AA) changes (the latter are in parentheses). The proportion of nonsynonymous (dN) to synonymous (dS) changes, numbers of transversions (Tv), and the proportion of transversions to transitions (Ts) are given, along with the evolutionary rate estimates. Totals are in the final rows.

In comparison, VHSV-IVa sequences (Table 1B) possessed 787 SNPs, of which 203 were nonsynonymous (0.26). The VHSV-I+III genogroup clade (Table 1C) contained 1292 SNPs, with 225 AA differences (0.17). Table 1 indicates that subgenogroup VHSV-IVb possessed the fewest transversions (Tv: VHSV-IVb = 42, VHSV-IVa = 131, VHSV-I+III = 254), tying with subgenogroup VHSV-IVa in the overall ratio of transversions: transitions (Tv/Ts = 0.166). The genogroup clade VHSV-I+III had a larger Tv/Ts ratio (0.197). The relative trends for individual genes for VHSV-IVb, -IVa, and -I+III are detailed in Table 1; these varied among the genogroups/subgenogroups.

Per isolate changes were examined for the more recent VHSV-IVb isolates. The average number of SNPs/isolate was 16.1, with 6.1 (0.38) AA differences (S3 Table). The C06NP group possessed the fewest SNPs, with a single non-synonymous change in *L*. Isolates from 2012–2016 contained the highest SNP proportions (averaging 27.9 SNPs, 9.5 AAs), ranging from 14 (M16RGa, 4 AAs) to 38 (E16GSa, 13 AAs). Isolates recovered from cell culture differed by a few SNPs from their original sequences; CellC03 diverged by four SNPs from C03MU and Cell16a–c were two, six, and eight differences from E16GSa, respectively (S3 Table).

**Fig 2:**
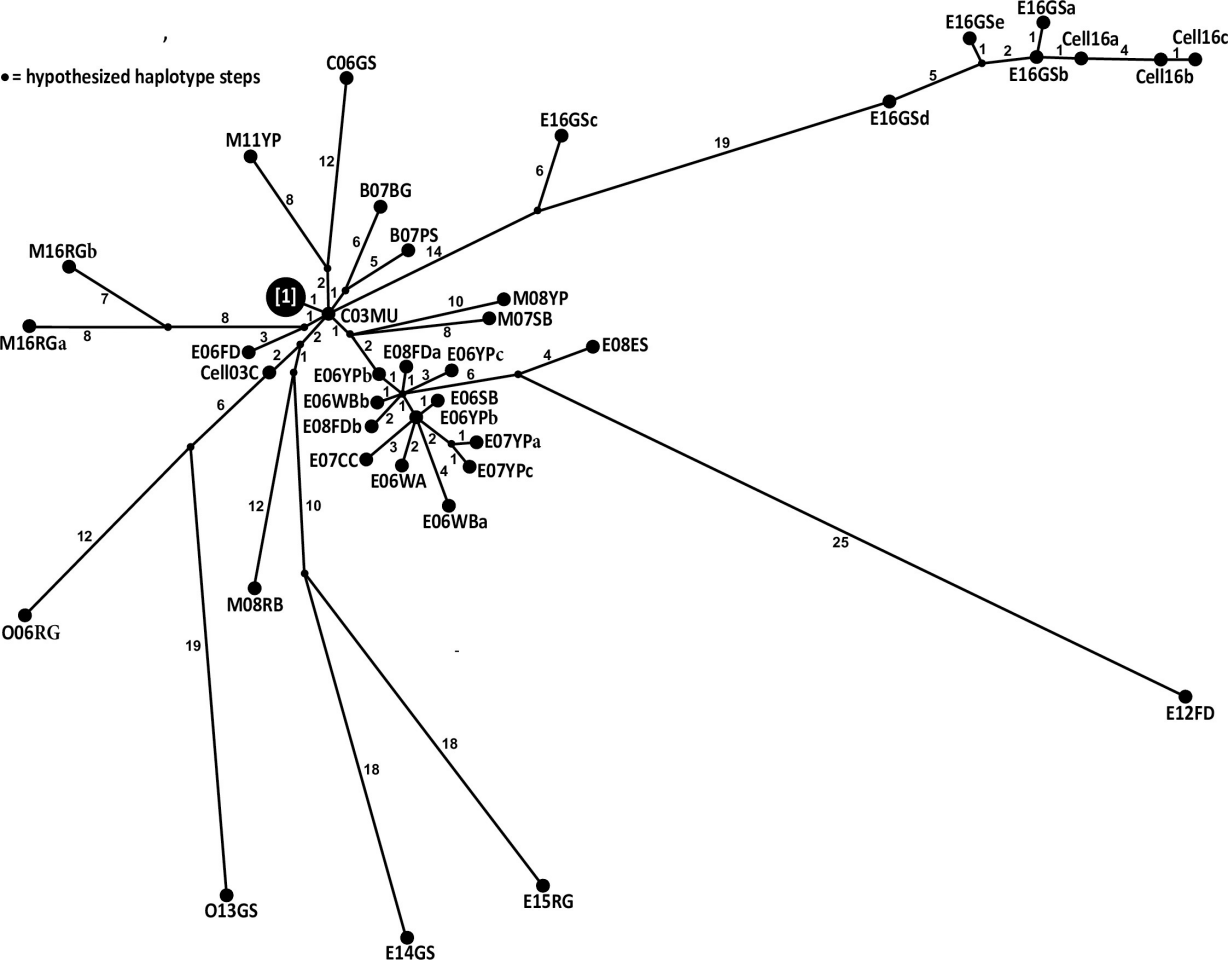
Haplotype network showing genetic relationships among 48 VHSV-IVb whole genomes determined by us with PopART freeware (http://popart.otago.ac.nz). Circles are sized according to haplotype frequency (number of isolates). Numbers inside parentheses designate NT differences between each haplotype and the original haplotype, C03MU*. Individual isolates sharing a common haplotype (labelled “1” here) include: C06NP, C06RB, C06SR, C06YP, C06FD, E06WBc, M08AMa,b, C08LEa,b, and C09MU); these are termed the C06NP group. Small, unlabeled black circles = hypothesized haplotype steps.

SNP changes are denoted on the Fig 2 haplotype network. The oldest and original isolate, C03MU, appears centrally located nearby a large cluster of isolates, including the C06NP group. Sequences from the 2006–2008 Lake Erie outbreaks cluster together, differing by 1–4 SNPs. E12FD radiates from this cluster by 31 additional SNPs. Both isolates from Budd Lake (B07BS, PS) form an individual branch, sharing one synonymous SNP in the *G-*gene. The Lake Ontario isolates (O06RG, O13GS0) cluster together [also see 45, 51], sharing 10 SNPs before diverging by 31 SNPs. Five shared changes are nonsynonymous: one each in *M* and *L* and three in *G*. Lake Michigan isolates appear scattered throughout the network, but are closer to C03MU, with the 2016 isolates differing by 15–16 NTs (eight shared). E16GSa–e and derived cell culture isolates form a distant, smaller cluster, sharing 14 SNPs before E16GSc diverges. The remaining seven isolates share 19 SNPs, with E16GSb being central (Fig 2).

SNPs distinguish isolates of subgenogroup VHSV-IVa (S1 Fig) and genogroup clade VHSV-I+III (S2 Fig). In the VHSV-IVa network, the two Japanese isolates are very distant (KRRV9822: 84 NTs, JFEh001: 253 NTs) from the nearest Korean isolate (KJ2008). Two identical isolates occur in VHSV-I+III, which were recovered one year apart in Norway (BV06048–52, FA281107). Seven VHSV-Ib isolates from Sweden (1998–2000) and a Japanese fish farm isolate (1996) cluster together, differing by 2–22 SNPs (S1 Fig).

### Evolutionary rates and selection

The fastest overall evolutionary rate (Table 1) has characterized the VHSV-IVa subgenogroup (2.01x10^-3^ substitutions/site/year), followed by subgenogroup VHSV-IVb (6.64x10^-5^) and the genogroup clade I+III (4.09x10^-5^). For VHSV-IVa, NCDS has evolved with the highest rate (7.79x10^-3^), followed by the *P-* (2.32x10^-3^) and *Nv-*genes (2.03x10^3^). For VHSV-IVb, the NCDS has appeared faster (1.40x10^-4^), followed by *Nv* (9.76x10^-5^) and *G* (8.51x10^-5^). The order for VHSV-I+III is: *Nv* (7.78x10^-5^), *M* (5.45x10^-5^), and *N* (4.59x10^-5^). Table 1 contains further details about the relative evolutionary rates of all genes for VHSV-IVb, VHSV-IVa, and VHSV-I+III. The evolutionary rates of genes were not consistent among the genogroups/subgenogroups (i.e., some genes evolved faster and some slower in each genogroup/subgenogroup).

We examined selection pressures for the VHSV-IVb, VHSV-IVa, and VHSV-I+III per each CDS (Table 2). Results showed that purifying selection has characterized VHSV-IVb’s *N-* (codon 313), *G-* (codon 342), and *L-* (six codons: 8, 119, 333, 460, 1284, and 1758) genes. For VHSV-IVa and I+III, FUBAR implicated purifying selection for all genes (Table 2B and 2C). In VHSV-IVb, one codon (*L*, 1758) indicated purifying selection and three codons implied diversifying selection–for the *G-* (103, 431) and *Nv*-genes (25). A VHSV-IVa codon reflected diversifying selection (*G*, 12), along with one for -I+III (*N*, 46). MEME analysis results inferred diversifying selection for VHSV-IVb (*G*, 431) and VHSV-I+III (*G*, 477) and at three different *L-*gene sites each for VHSV-IVa (147, 593, 1154) and VHSV-I+III (112, 474, 1012); none of these matched among the genogroups.

**Table 2:** Positive (diversifying) or negative (purifying) selection pressures on individual codons for genes from whole genome sequences, determined by FUBAR (fast, unconstrained Bayesian approximation) and MEME (mixed effects model of evolution) analyses [70, 71] for (A) VHSV-IVb, (B) VHSV-IVa, and (C) VHSV-I+III (combined).

**A. VHSV-IVb**			
**Gene**	**FUBAR diversifying (*pp* > 0.95)**	**FUBAR purifying (*pp* > 0.95)**	**MEME diversifying (*p*<0.05)**
***N***	0	1 (313)	0
***P***	“”	0	“”
***M***	“”	“”	“”
***G***	2 (103, 431)	1 (342)	1 (431)
*Nv*	1 (25)	0	0
***L***	0	6 (8, 119, 333, 460, 1284, 1758)	“”
**B. VHSV-IVa**			
*N*	**0**	**8 (none in common)**	**0**
***P***	“”	1 (113)	“”
***M***	“”	2 (145, 166)	“”
***G***	1 (12)	5 (28, 75, 157, 216, 301)	“”
***Nv***	0	1 (24)	“”
***L***	“”	30 (none in common)	3 (147, 593, 1154)
**C. VHSV-I+III (combined)**			
***N***	**1 (46)**	**20 (none in common)**	**0**
***P***	0	8 (none in common)	0
***M***	0	1 (160)	0
***G***	0	19 (none in common)	1 (477)
***Nv***	0	3 (56, 96, 109)	0
***L***	0	447 (matches -IVb at 1758)	3 (112, 474, 1012)

None of the codons found in VHSV-IVa or VHSV-I+III matched the codons that were under selection for VHSV-IVb. Results of seven or more codons under selection not displayed. *pp* = posterior probability.

### Evolutionary relationships

**Fig 3:**
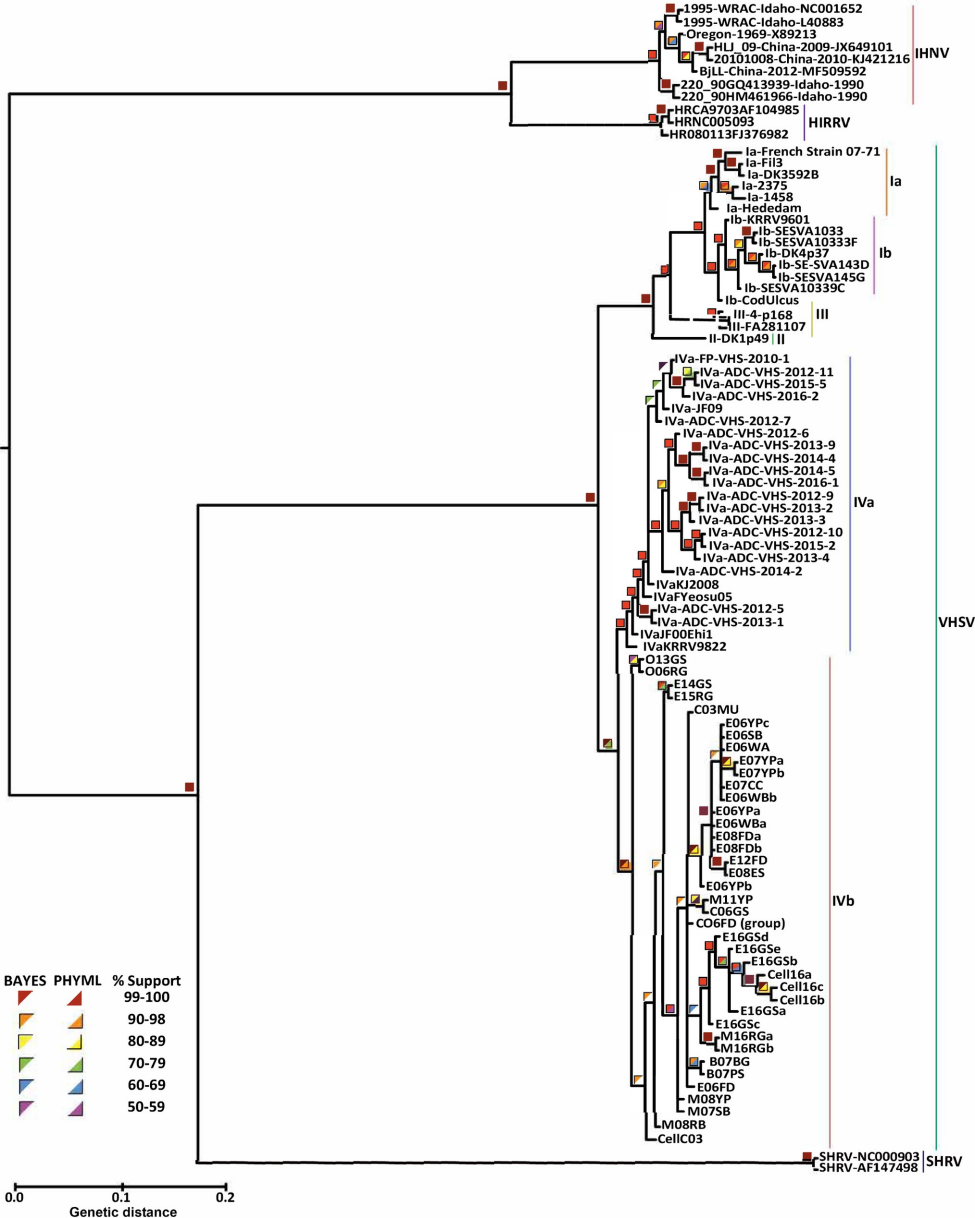
*Novirhabdovirus* phylogenetic tree, based on aligned whole genome sequences analyzed here (see S1 and S2 Tables) using maximum likelihood and Bayesian analyses. Colored squares designate support values, top left half = Bayesian posterior probabilities, bottom right = 500 bootstrap pseudoreplicates. Tree is rooted to the *Snakehead novirhabdovirus* (SHRV, GenBank: AF147498).

The *Novirhabdovirus* phylogenetic tree determined here (Fig 3) defines each species with 1.00 posterior probability (*pp*) and 100% bootstrap support (bs). The tree shows that IHNV and HRRV are sister species, whose clade comprises the sister group to VHSV+SHRV. The 79 genomic VHSV sequences analyzed form two primary clades: European genogroups VHSV-I–III and North American/Asian VHSV-IV.

Within the VHSV I–III genogroup clade (Fig 3), the single VHSV-II isolate analyzed (II-DK1p49; GenBank: KM244767) is located basally to all of the others (i.e., to the other VHSV-I and -III isolates sequenced). The VHSV-Ia isolates are monophyletic, as well as the VHSV-Ib isolates, with VHSV-Ib-CodUlcus (GenBank: Z93414) appearing basally located, and divergent from the main group of the VHSV-Ib clade. Our phylogenetic results (Fig 3), also strongly support the monophyly of the VHSV-IV genogroup (1.00 *pp* and 100% bs), as well as the monophyly of its subgenogroups, VHSV-IVa and VHSV-IVb (1.00 *pp* and 100% bs, respectively).

**Fig 4:**
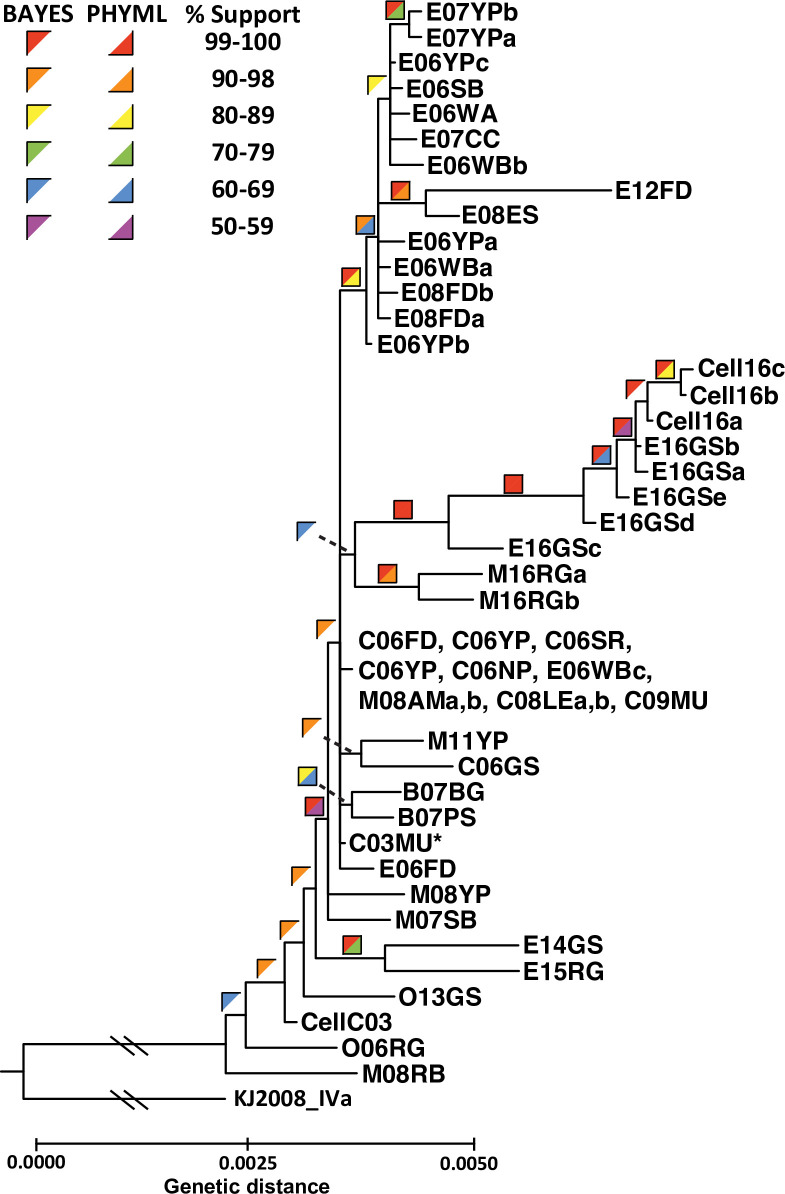
VHSV-IVb phylogenetic tree of aligned VHSV-IVb whole genome haplotypes, from our maximum likelihood and Bayesian analyses. Colored squares = support values, top left half = Bayesian posterior probabilities, bottom right half = 1450 bootstrap pseudoreplicates. Hashes represent cropped region for visualization. * = original VHSV-IVb isolate. Sequences from cell culture amplified isolates are labeled (E16GSa: Cell16a; E16LB:Cell16b and Cell16c). The tree is rooted to subgenogroup VHSV-IVa (GenBank: JF792424).

The VHSV-IVb phylogenetic tree based on our whole genome sequences (Fig 4) contains several inner clades, with isolate M08RB being located basally (weakly supported), followed by O06RG, CellC03, and O13GS, respectively (0.60–.90 *pp*/<50% bs). Two Lake Erie isolates from 2014–2015 (E14GS, E15RG) cluster together (1.00 *pp*/74% bs), followed by two from Lake Michigan in 2007–2008, which are located before the main cluster containing the remaining 39 isolates (including the original isolate–C03MU). Within the major group are two clades of two isolates each (B07BG/B07PS: 0.80–.89 *pp*/66% bs; M11YP/C06GS: 0.80–0.89 *pp*/<50% bs) and two larger clades. The first larger clade incorporates the 2016 isolates (0.60–0.69 *pp*/<50% bs), which further is subdivided into two groups that separate a group of two Lake Michigan isolates (1.00 *pp*/98% bs) from a group of five Lake Erie isolates; the latter also contains the three cell culture derivatives (1.00 *pp*/100% bs). A second well-defined clade (1.00 *pp*/83% bs) contains14 isolates, including most early to middle (2006–2008) Lake Erie genotypes, along with a very distinct 2012 isolate (E12FD). That 2012 genotype is the most genetically distant, comprising the sister taxon to E08ES (1.00/94%); the two are linked by four shared SNPs and 2 AAs (*M-*gene: one SNP, one AA; *L*: two SNPs, one AA; NCDS: one SNP). Isolates E12FD and E08ES share an additional five SNPs, including two in the *N-*gene (two AAs), and two in the *L-*gene (one AA).

### Differences in cytopathogenicity and immune response

A series of cell-based studies were used to determine whether sequence variations impacted viral function: SRB assays to assess cytopathogenicity, virus yield assays to estimate viral production, antiviral assays to assess IFN production, and RT-qPCR to determine levels of IFN and VHSV-IVb mRNA production. Sequencing confirmed each isolate’s identity and revealed changes acquired during cell culture propagation. The cell culture amplified control–CellC03 –differed from C03MU by four NTs and two AAs; these were: *M* (2312, NT:C–T, AA:T–I) and *G* (3397, NT:G–A, AA:K–D; 4007, NT:C–G, AA:G–G; 4394 NT:G–A, AA:V–V). Cell16a–c differed by 34–38 SNPs (13–14 AAs) from C03MU (S3 Table). From E16GSa, Cell16a differed by two NTs: *N* (737, NT:C–A, AA:T–T) and *G* (4117, NT:G–A, AA:D–N), Cell16b differed by four additional changes (six total) in *L* (6962, NT:C–A, AA:A–E; 7038, NT:G–A, AA:E–E; 7047, NT:T–C, AA:C–C; 7647, NT:T–C, AA:H–H), and Cell16c had one additional NT difference (one total) in *L* (6456, NT:C–T, AA:L–L).

**Fig 5:**
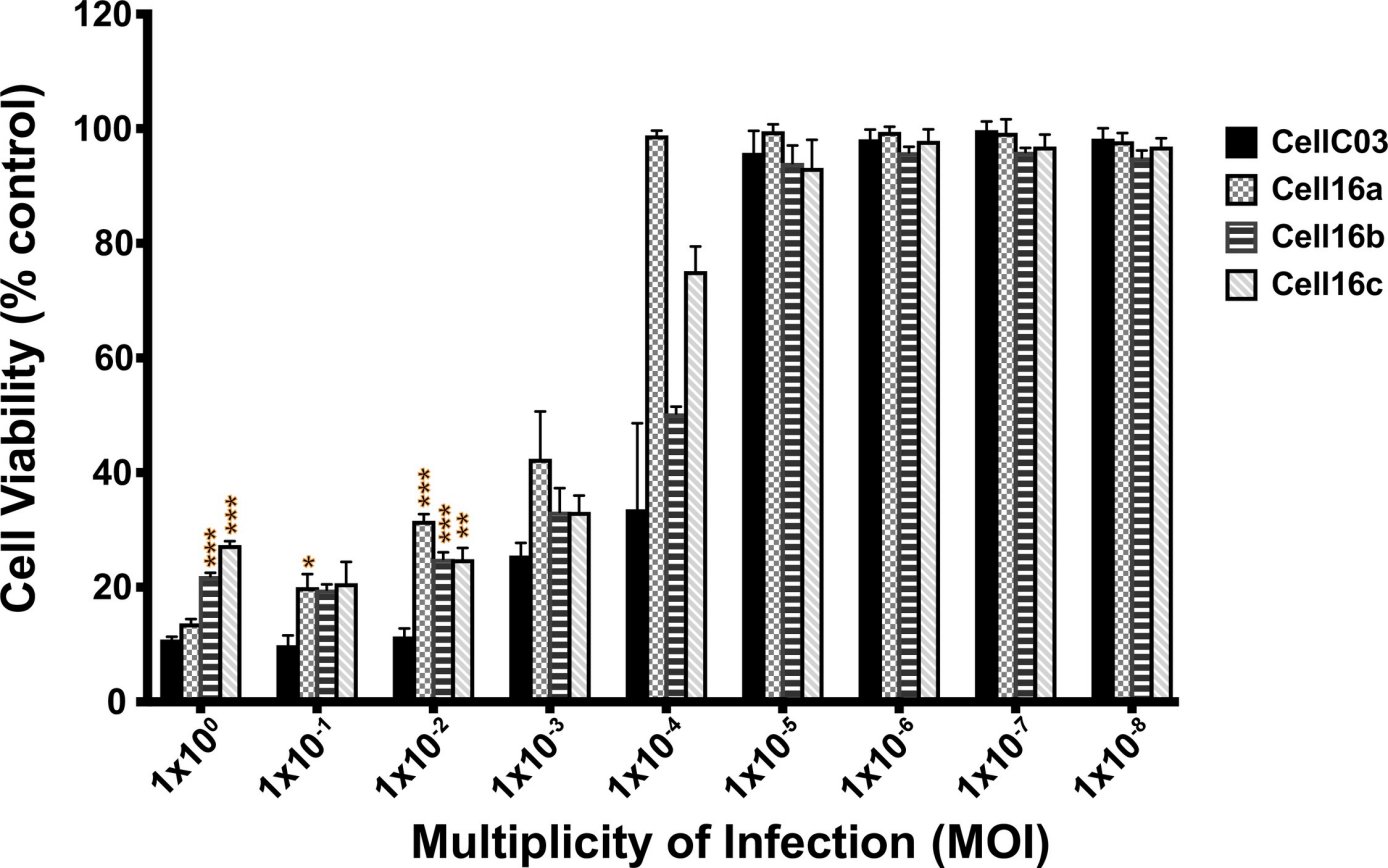
Host cell viability at 96 hpi with VHSV-IVb isolates. Residual cell viability was measured using the SRB assay in EPC cells infected with a series of MOIs. Standard error bars are representative of at least three independent experimental replicates. Statistical significance is shown: *p<0.05; **p<0.01; ***p<0.001.

**Fig 6:**
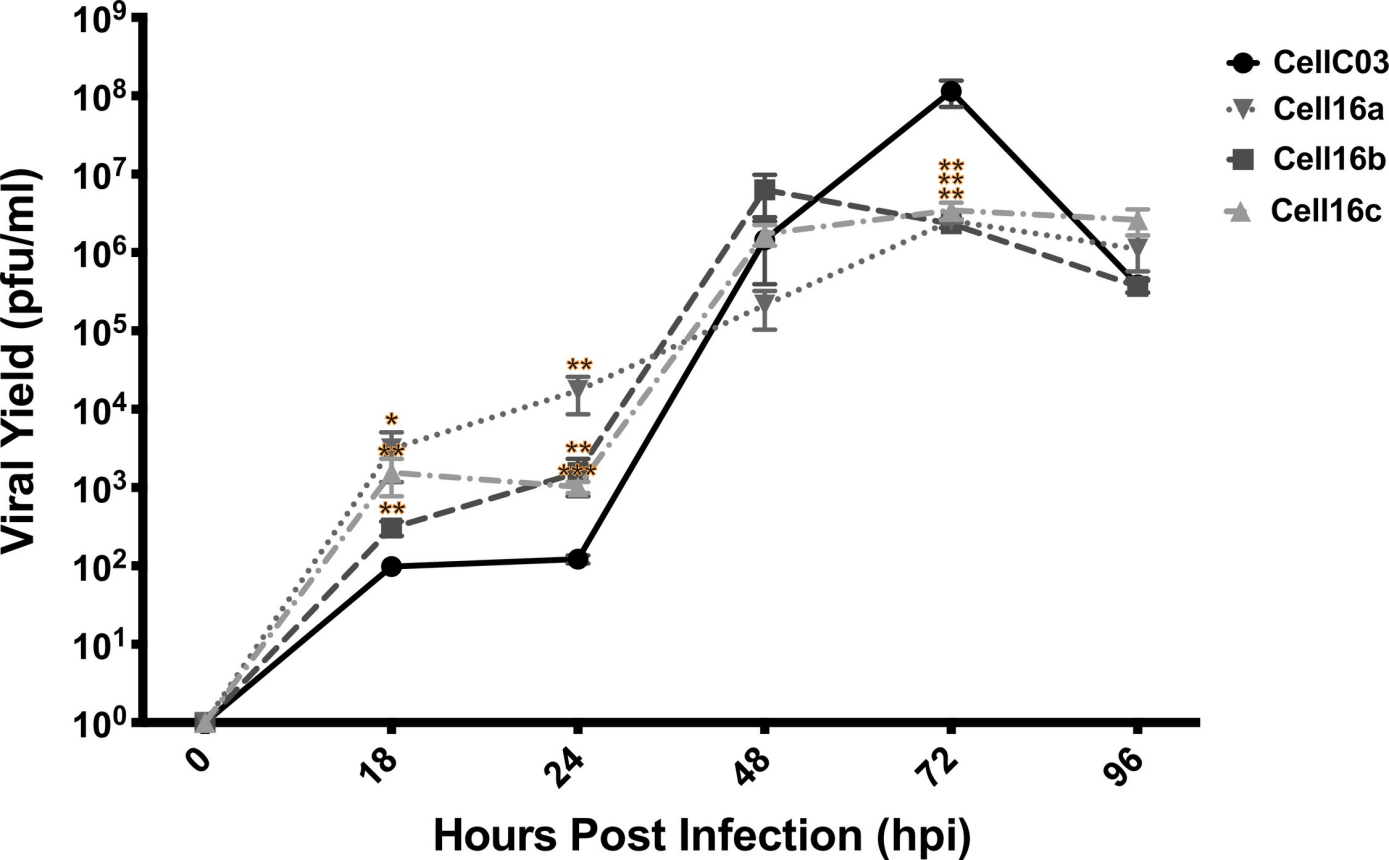
Viral yield assay comparison of infectious viral particles produced (pfu/ml) in wild type (CellC03) and three 2016 VHSV-IVb samples, in BF2 cells 96 hpi, following exposure to media from collection time post infection. Standard error bars are representative of at least three independent experimental replicates. Statistical significance is shown: *p<0.05; **p<0.01; ***p<0.001.

SRB staining examined CPE elicited at different MOIs across the four isolates. The 2016 isolates induced less CPE than CellC03 when tested at MOIs >1x10^-3^ (Fig 5). Although not statistically different across all isolates and at all dilutions, the 2016 viral isolates tested were generally less cytotoxic than CellC03. Consistent with this observation, viral yield assays demonstrated that at 72 hpi, CellC03 produced significantly more virus than the 2016 isolates (Fig 6), yielding nearly 100-fold more infectious particles as compared to Cell16a–c. Although viral yield was not different by 96 hpi, it is important to note that cells infected with CellC03 were largely dead by that time (see Fig 5), and thereby unable to continue producing virus.

### IFN production and expression of VHSV and IFN mRNAS

**Fig 7:**
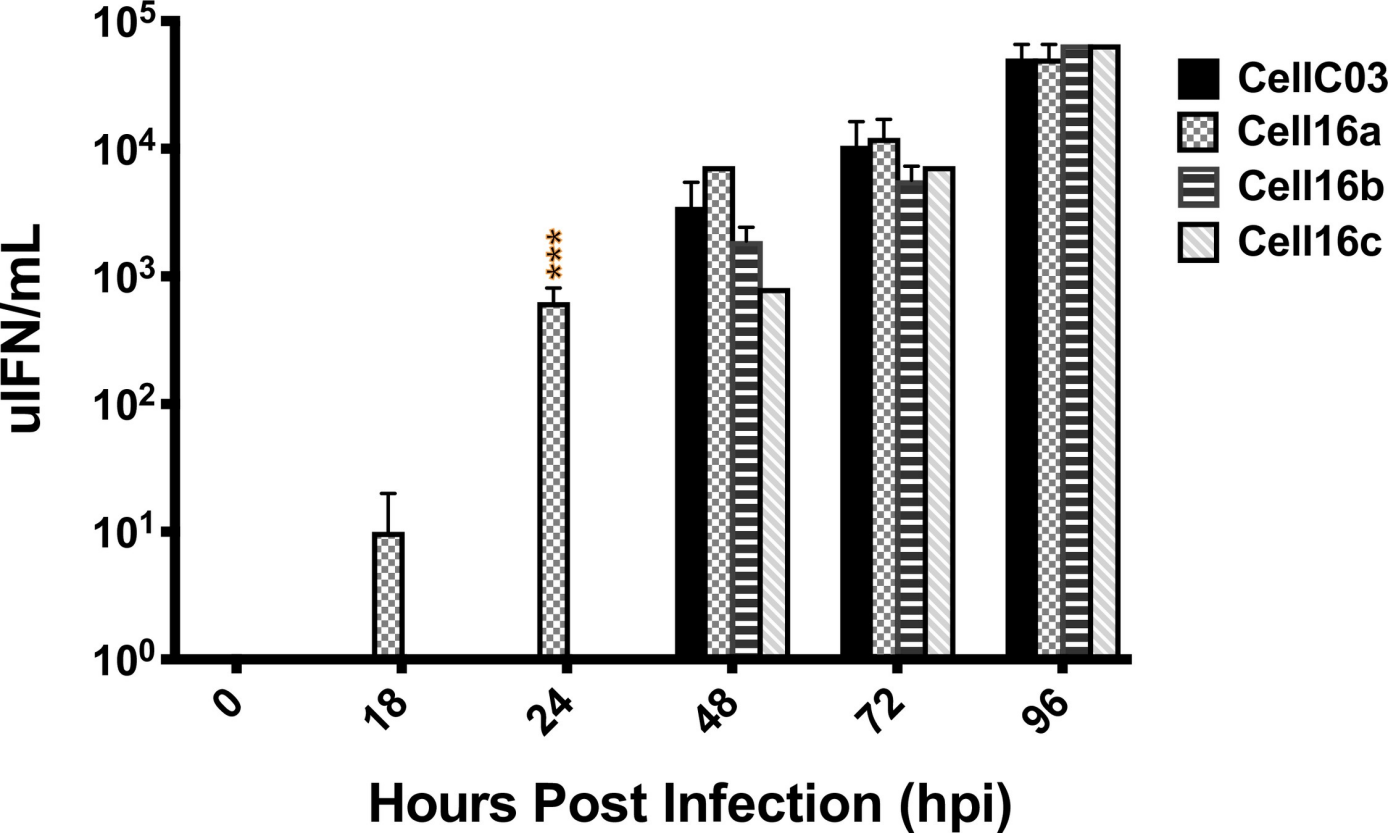
Antiviral assay comparison of host IFN suppression between reference (CellC03) and three more recent VHSV-IVb isolates, in EPC cells 96 hpi following exposure to UV irradiated media collected at the above time points. Values are quantified as the number of antiviral units (uIFN) per ml. Standard error bars are representative of at least three independent experimental replicates (some are too small to visualize). Statistical significance is shown: *p<0.05; **p<0.01; ***p<0.001.

**Fig 8:**
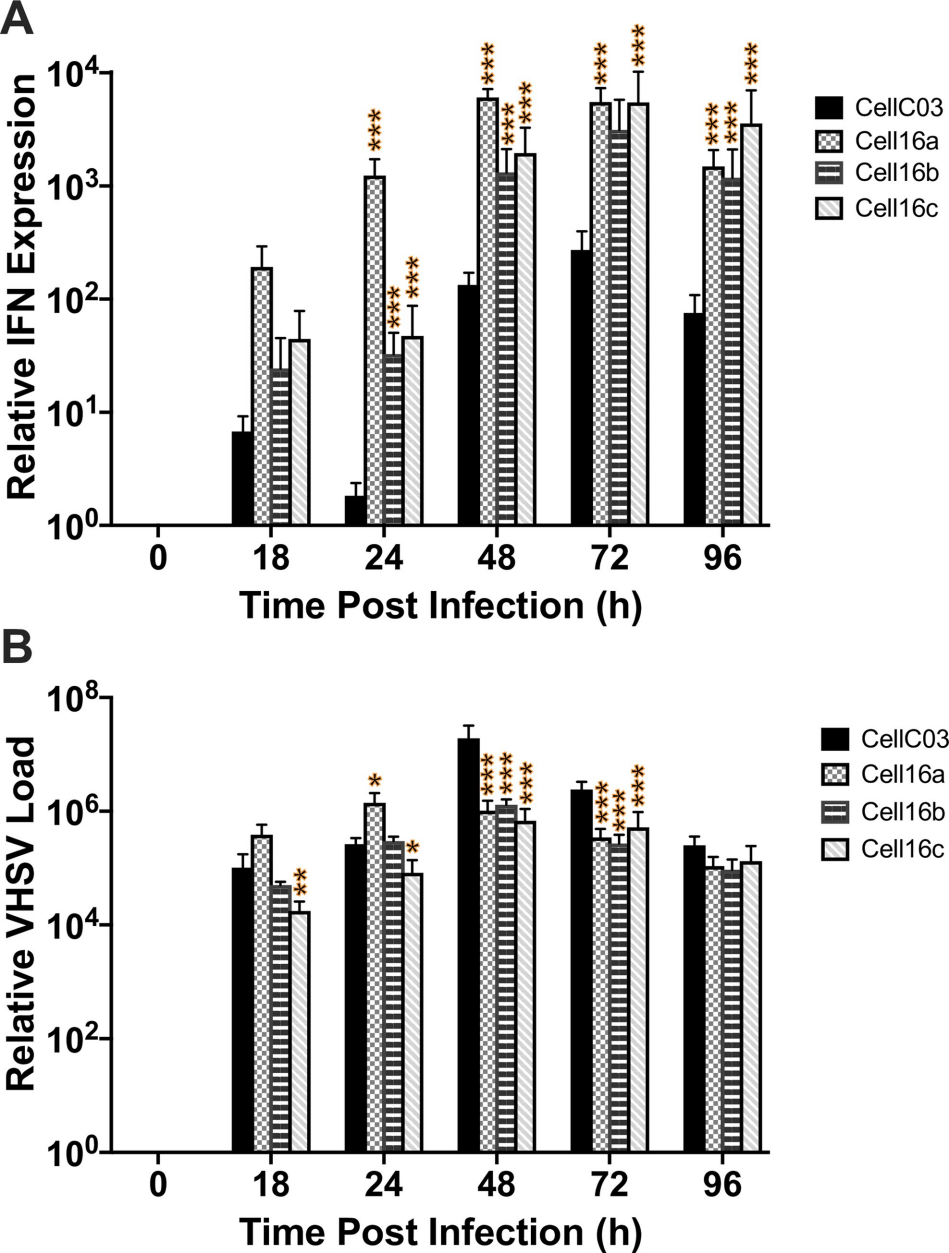
Measures of host immune response and viral RNA produced. RT-qPCR comparisons between reference (CellC03) and three 2016 VHSV-IVb isolates for: (A) EPC IFN gene transcription and (B) virus detected in samples at each time point. Data were normalized to β-actin mRNA levels. Standard error bars are representative of at least three independent experimental replicates. Statistical significance is shown: *p<0.05; **p<0.01; ***p<0.001.

IFN production was measured in an antiviral bioassay, which revealed earlier onset of antiviral immune response in cells infected with Cell16a, yielding significantly more IFN at 24 hpi than CellC03 (Fig 7). All isolates produced comparable IFN activities after 24 hpi. Since IFN antiviral bioassays are relatively insensitive to minimal changes, we extended the previous studies by assessing IFN mRNA synthesis in infected cells with RT-qPCR. IFN mRNA was expressed significantly more in Cell16a–c, compared with the CellC03 control across all time points beyond 24 hpi (Fig 8). These data suggest significant difference in ability of the 2016 isolates to induce cellular IFN expression, or significant reduction in their suppression of IFN expression. Consequently, by 48 hpi, CellC03 produced the highest overall amount of viral RNA at 48 hpi, which continued until 96 hpi, by which point cell death in all cultures led to reduced viral expression. All 2016 isolates produced similar amounts of viral RNA.

## Discussion

### Evolutionary patterns

We analyzed 48 field-collected isolates of VHSV-IVb for the largest *Novirhabdovirus* genomic investigation to date. The greatest proportion of SNPs occurred in the NCDS (4.3%) and the *Nv*-gene (3.8%). For the *Nv-*gene, none of the isolates we sampled had >two SNPs, suggesting that sequence conservation is important. An experimental study on *Nv* function indicated that mutations at codon positions 36, 39, and 41 resulted in greater host immune suppression [28]; however, our sequences lacked SNPs at those locations. Since *Nv* is not essential for viral replication [19], its SNP variations may be more tolerable for the virus. Large proportions of SNPs likewise characterized the NCDS for genogroups VHSV-IVa and VHSV-I+III, illustrating sequence flexibility. There is no evidence that NCDS regions are transcribed or play a functional role in any rhabdovirus [2], thus these SNPs likely are selectively neutral (or relatively so).

Diversifying selection was indicated for two VHSV-IVb genes: *Nv* and *G*. Unlike our findings, a study of four VHSV-IVb genome sequences (C03MU, O06RG, O13GS, and E14GS) by Getchell et al. [44] did not uncover diversifying selection for either the *Nv-* or *G*-genes. Getchell et al. [44] discerned variation in two *N-*gene codons and one codon each for *M* and *L*, which were unsupported in our investigation. We found evidence of purifying selection acting on single *N-* and *G-*gene codons, and on six codons in *L* in our VHSV-IVb analyses. Thus, fewer codons appeared to be under purifying or diversifying selection in our study than in Getchell et al.’s [44] findings. None matched between the two analyses, with ours being a larger, more robust dataset. A more recent study by Getchell et al. [56] of nine more VHSV-IVb whole genome positives, including five from the 2017 Cayuga Lake, NY outbreak and another four from a 2017 round goby kill at Long Point State Park, NY), noted 379 SNPs, with 123 being non-synonymous (33.4%). Those values also are higher than ours and might again reflect proof-reading for sequence error, sampling size, or mutation rate variation. This difference between study results should be evaluated further.

Purifying selection was the major evolutionary force discerned across all VHSV genogroups, subgenogroups, and samples. Our study identified ≥one codon per gene under negative selection, for VHSV-IVa and -I+III. No NT sites under selection were in common between VHSV-IVb and the other genogroups, with one exception: codon 1758 in the *L-*gene for VHSV-IVb and VHSV-I+III alike. Our dN/dS ratio further indicated purifying selection, with values being >0.5 across all genes and VHSV genogroups. Other investigations have found that purifying selection regulates VHSV evolution [75, 76], as well as in other rhabdoviruses [77] and RNA viruses in general [78]. He et al. [76] compared dN/dS ratios for individual genes among VHSV genogroups, with all six genes having lower dN/dS than found with our data. However, most of the VHSV-IVb (N = 45) and VHSV-IVa (*N* = 18, from [79]) sequences used in our analyses were unavailable to He et al. [76].

Positive selection was indicated for changes in three *G*-gene codons: two in VHSV-IVb and one in VHSV-IVa. Positive selection on viral genes constitutes evidence that supports the host-pathogen “arms race”, which typically suppresses host immune responses [50]. Abbadi et al. [75] examined 108 VHSV-Ia full *G*-gene isolates, discerning positive selection on two codons (258, 259). He et al. [76] also elucidated positive selection for VHSV-I at codons 258 and 476, but not for 259. Our codon results did not match either of those studies.

We found that overall evolutionary rates across the entire VHSV genomes were slower than were estimates based on partial gene sequences [9, 14]. These differences stem from our present use of more samples and complete genome sequences that included conserved areas, which previously had been omitted.

Among the VHSV genogroups examined here (VHSV-IVa, VHSV-IVb, and VHSV-I+III), VHSV-IVa exhibited the fastest rate of 2.01x10^-3^. He et al. [76] estimated a rate of 5.60x10^-4^ for 48 VHSV-IVa *G*-gene sequences, which was slower than the rate we calculated for the VHSV-IVb *G-*gene of 1.02x10^-3^. Our inclusion of recently published diverse VHSV-IVa sequences from Korea (collected 2012–2016) [79] may have increased this rate estimate, as those isolates differed by >20 NTs. Just two complete sequences of VHSV-IVa were from Japan, which were even more different than the Korean isolates. This suggests that the Korean isolates are diverging from the Japanese VHSV-IVa isolates, but additional VHSV-IVa genomes from Japan are needed for more conclusive analyses. Subgenogroup VHSV-IVa first was detected along the North American Pacific Coast [32–34], but no whole-genome sequences then were available. All VHSV-IVa Korean isolates were obtained from aquaculture; such transportation of infected fishes may introduce VHSV to naïve fishes and different environmental conditions, which can enhance adaptation and dramatically alter the virus, as observed in IHNV [80] and HIRRV [81].

We calculated that VHSV-I+III has evolved at an overall rate of 4.09x10^-5^, similar to that determined here for VHSV-IVb. Previous *G*-gene rate estimates [76] for VHSV-I (5.57x10^-4^) and -III (1.63x10^-3^) using fewer complete gene sequences were faster than ours here (4.54 x 10^−5^). Evolution of subgenogroup VHSV-Ia had been estimated at 1.74x10^-3^ (*N* = 34) [23] to 7.3x10^-4^ from 108 *G*-gene Italian isolates [75]. Our combination of the European genogroups may have yielded our slower rate. Despite these variations, the above estimates are within the known range of evolutionary rates for RNA viruses [82].

### Phylogenetic patterns of Novirhabdoviruses

The phylogeny we obtained from the whole genome analyses (Figs 2 and 3) is congruent with results from prior partial gene studies [9, 14, 18]. Two main sister groups characterize the novirhabdoviruses: IHNV+HIRRV and VHSV+SHRV, with VHSV+SHRV diverging first from the common ancestor. This relationship is congruent with Kurath’s (2012 [18]) analysis of complete *N-*gene sequences. Two studies examined rhabdoviruses using partial *L-*gene sequences [83, 84], which likewise supported our sister group pairing of IHRV+HIRRV. Our phylogenetic consensus tree yielded 100% ML and Bayesian support for these relationships, whereas Kurath [18] found just 76% support for the SHRV+VHSV clade using the *N-*gene. Thus, our analysis of the entire genome using a larger number of isolates significantly increased confidence in resolving *Novirhabdovirus* evolutionary relationships.

Phylogenetic results from whole genome sequences support the known classification of VHSV genogroups and subgenogroups (as indicated on Fig 3), congruent with prior analyses of *G*-gene sequences by Dale et al. [85] and Ghorani et al. [86]. Subgenogroup VHSV-Ia infects freshwater fish hosts [23], whereas both VHSV-III and -Ib characteristically have marine fish hosts [85]. All three are capable of infecting the rainbow trout (*Oncorhynchus mykiss*), which spends portions of its lifecycle in both environments. Our results indicate that VHSV-Ia, -Ib, and -III are each monophyletic. VHSV-II is located basally, comprising the sister group to VHSV-I+-III, and VHSV-III is the sister group to VHSV-I.

### Phylogenetic patterns of VHSV-IVb

Our VHSV-IVb phylogenetic tree contains several clades of closely related isolates, which correspond to their sampling locations and years. We resolve an in-depth view of the virus in Lake Erie, where a large portion of our samples were collected. The inner-most branches of the phylogeny indicate that at least two separate infections led to the major outbreaks, since one of the 2006 isolates (E06FD) appeared more closely related to C03MU and the Lake St. Clair outbreak samples.

The differentiated Lake Erie clade from 2006–2008 and 2012 suggests its separate evolutionary history from the two other (2014–2015 and 2016) Lake Erie clades. Getchell et al. [56] found that the Cayuga Lake 2017 outbreak isolates were more closely related to Lake Erie isolates. Our study shows that broad diversification characterized the 2006 outbreak, which continued along that trajectory in 2007–2008, with minor variants. The lone 2012 sample, although evolutionarily distant, appears more closely related to the E06–08 clade, than to Lake Erie samples from subsequent years. E12FD forms a sister clade with E08ES, sharing an additional four AA changes in the coding regions of *N*, *P*, and *L*. These AA differences possibly provided some advantage or lacked deleterious effects, since the substitutions remained conserved for four years. Although most of that clade was recovered from Percidae hosts, several of its isolates were in freshwater drum (*Aplodinotus grunniens*). In addition to the 2006 and 2008 Lake Erie cases [12], freshwater drum die-offs were reported in 2005 from Lake Ontario [11], which appeared more closely related to E06FD.

Both of our 2007 samples from the inland Budd Lake (Harrison, MI) form a clade, which is placed near C03MU on the phylogenetic tree. That geographic region is a highly urbanized area containing the largest number of registered anglers in the state [87]; thus, anglers traveling to Budd Lake may have unknowingly transported VHSV, perhaps via live bait [9, 14, 88].

The 2014–2015 Lake Erie VHSV-IVb genotypes differed from one another but share a sister relationship branching from the main part of the phylogeny, located closer to C03MU. Genotypes E14GS and E15GS shared the host species of gizzard shad and round goby with the 2016 isolates, but were genetically distant from the latter. The 2016 genotypes form a clade, which is linked by SNPs as shown in the genotype network. Interestingly, these occurred in different host species, with the E16 isolates exclusively recovered from gizzard shad, and both M16 isolates from round goby. Other studies have identified potential evolutionary radiation of VHSV-IVb in the round goby host, particularly in Lake Ontario [39]. There have been relatively few VHSV-IVb-positive round goby samples detected in Lake Michigan, despite reported mortality during the 2008 outbreaks [16]. The invasive round goby has been implicated in spreading the virus [89], often is used as bait, and is transported among water bodies by anglers [90]; these factors suggest its likely role as a primary vector.

Other VHSV-IVb-positive isolates were recovered from Lakes Michigan and Erie in 2016, whose viral titers were too low for genome sequencing [51, 57], further indicating that infection levels differ spatially and temporally. One of the first 2006 outbreaks intensely affected the Lake St. Clair gizzard shad population [16], as well as its 2017 outbreak (G. Whelan, Michigan Department of Natural Resources, personal communication 2017). Gizzard shad populations frequently experience large die-offs from cold weather [91], water temperature fluctuations, and/or spawning activities [92], coinciding with the optimal temperature range of VHSV-IVb (12–18°C [93]; 10–14°C [94]. In addition to the 2016 isolates, VHSV-IVb-positive gizzard shad also were found in Lakes St. Clair, Erie, and Ontario dating back to 2006; however, only its 2016 isolates were closely related to one another. Possible host specialization should be examined in future investigation.

All but one of our identical VHSV-IVb sequences, comprising the C06NP group, were collected from Lake St. Clair over a five–year span. This consistency suggests that the C06NP genome may have been prevalent and locally adapted to environmental conditions and host species. C06NP differed from C03MU by a single AA in the *L-*gene, and was found in six different fish host species and the sole two known invertebrate host species: leech and amphipod (*Diporeia* spp.) [95, 96]. Yusuff et al. [97] detected no effect on host specificity when swapping the *L*-genes between C03MU and VHSV-Ia DK-3592B. *L-*gene variants appear to differ in their optimal temperature range, since swapping *L* from VHSV-IVa into VHSV-IVb led to increased virus at higher temperatures, which did not occur in VHSV-IVa [98]. The single AA difference between the C06NP group and C03ML was not shared with VHSV-IVa; instead C03ML and VHSV-IVa were identical at that NT, meriting future examination.

Our accompanying analyses of population divergence patterns using the entire VHSV-IVb genome [51] discerned similar spatial and temporal patterns as found in our prior partial *G-*gene analyses [14, 15, 51]. Those results showed that increased divergence has characterized the later time period (2012–present), as compared to the early (2003–2007) and middle (2008–2011) time periods of VHSV-IVb in the Great Lakes. During the later time period, VHSV-IVb has occurred more sporadically in smaller, localized outbreaks, which may reflect increased host population immunity and resistance, as predicted by the “Red Queen hypothesis” [48, 49]. Likewise, viral phage Φ2 showed greater genome divergence when exposed to changing hosts versus consistent ones, particularly in genes associated with host immune suppression [99]. On the host side, Alves et al. [100] described genomic changes in immune-related genes of wild rabbit populations following 60 years of exposure to Myoxma virus (*Leporipoxvirus*) (MYXV).

### Differences in cytopathogenicity

After 24–72 hpi, the 2016 VHSV-IVb isolates produced less RNA and induced higher IFN transcription, compared to the reference 2003 strain (CellC03) (Fig 8). Although viral yields did not significantly differ, by 96 hpi CellC03 produced more active viral particles (Fig 6) and was more cytotoxic at higher MOIs (Fig 5). The genetic changes seen between CellC03 and Cell16a–c may have affected the ability to suppress host response and cause cellular damage. These may contribute to the virus appearing less virulent over time. A study by Cano et al. (2016) of VHSV-I genetic variants discerned differences in gene expression and inhibition of *Mx* proteins, which are key components of the antiviral state induced by interferons; results thus indicated effects on pathogenicity [101]. A recent investigation by Baillon et al. (2020) of VHSV-I supported importance of the *Nv-* and *N-*proteins in virulence, with three major gene virulence markers identified [102].

Cell16b–c (both isolated from E16LB) differed by a single *L-*gene NT and from Cell16a (isolated from ESG16a) by four *L-*gene NTs (one AA). Single nonsynonymous changes can have functional consequences for VHSV. Notably, Chinchilla and Gomez-Casado [28] denoted the function of SNPs in the *Nv-*gene by inducing mutations in VHSV-Ia strain FR07-71. Ke et al. [42] found that when four AA *M*-gene mutations were introduced to C03MU, the virus was less able to suppress the transcription of host immune defenses. These data imply that even subtle changes can lead to altered gene function and modulate viral phenotypic characteristics.

It appears that the oldest VHSV-IVb isolate, C03MU, has been the most virulent that we know of to date. More recent isolates from 2006–2016 showed reduced virulence, potentially resulting from virus–host coevolution. Other studies observed similar trends. For example, Imanse et al. [43] examined vcG001 (C03MU) and vcG002 (not included in our analysis), finding faster growth but lower titers in cells exposed to vcG002. These had similar levels of viral RNA, suggesting that vcG002 was less efficient at producing infective particles. Getchell et al. [44] also found reduced viral load in O06RG, O13GS, and E14GS. In our results, viral RNA production and virulence of Cell16a–c likewise did not significantly differ from CellC03, as evidenced by lower peak virus production in the viral yield assay. Reduced virulence over time has characterized MYXV in Australian rabbits [103], human papillomaviruses [104], and RABV in hyenas [105], leading to a longer infectious period that may aid spread of the virus. Such pattern may similarly characterize fish viruses and VHSV-IVb, meriting further work.

From our findings, we are unable to determine the mechanism underlying the observed reduction in virulence for the VHSV-IVb 2016 isolates that we examined. Reduced virulence might indicate less ability to suppress the host immune response, or it could be that the stronger immune response is the consequence of reduced virulence itself, allowing infected cells more time to produce antiviral factors. Some investigations have observed large impacts on virulence from single nucleotide changes in VHSV-I [29] and Vesicular stomatitis virus (VSV; Rhabdoviridae) [106]. A more recent study sequenced 55 genomes from VHSV-Ia isolates and identified 38 single amino acid polymorphisms distributed throughout the genome. By combining those data with mortality data from experimental challenges in rainbow trout, the authors identified several changes that correlated with virulence, either low or high. Those studies suggested that mutations at various locations throughout the genome can impact virulence, including changes in genes not previously associated with high or low virulence phenotypes [107]. Thus, additional studies assessing the role of mutations on a genome-wide basis are justified as a means of understanding natural changes in virulence and transmission.

## Conclusions

As VHSV-IVb nears the end of its second decade of evolution in the Great Lakes region, the virus has continued to evolve genomically, genetically, and phenotypically in virulence to its hosts. To date, subgenogroup VHSV-IVb has contained fewer SNPS than has characterized subgenogroup VHSV-IVa or the genogroup clade VHSV-I+III. Subgenogroup VHSV-IVa appears to have evolved the fastest, with the rates of VHSV-IVb and VHSV-I+III being similar to each other. Our *in vitro* studies show that more recent isolates of VHSV-IVb exhibit reduced virulence, consistent with other findings and field observations. Overall, VHSV-IVb may continue to threaten fish populations in the Laurentian Great Lakes region, and other waterways in continental North America, as it continues to evolve. These patterns suggest an overall trend towards persistence at relatively low levels and lesser virulence in host populations over time, in concert with predictions of the Red Queen hypothesis.

## Supporting information

S1 Fig Haplotype network showing genetic relationships among 24 VHSV-IVa whole genomes determined here using POPART freeware. Circles sized according to haplotype frequency among isolates. Numbers inside parentheses designate NT differences between each node, unlabeled black circles = hypothesized haplotype steps. Year and location of isolation are below isolate names.
(TIF)

S2 Fig Haplotype network showing genetic relationships among VHSV-Ia, VHSV-Ib, and VHSV-III whole genomes analyzed and created by us using PopART freeware. Circles sized according to haplotype frequency among isolates. Numbers inside parentheses designate NT differences between each node. Small, unlabeled black circles = hypothesized haplotype steps. Year and location of isolation are below isolate names.
(TIF)

S1 Table VHSV isolates for whole genome analysis. [25, 41, 44, 51, 55, 79, 97, 98, 108–118].
(DOCX)

S2 Table Additional *Rhabdovirus* sequences used in phylogenetic trees. [21, 109, 119–126].
(DOCX)

S3 Table Single nucleotide polymorphisms (SNPs) and nonsynonymous changes per individual isolates. * = Group, includes C06NP, C06RB, C06SR, C06YP, C06FD, E06WBc, M08AMa,b, C08LEa,b, and C09MU.
(DOCX)
