# Supplementary figures and images for: Genomic and immunogenic changes of Piscine novirhabdovirus (Viral Hemorrhagic Septicemia Virus) over its evolutionary history in the Laurentian Great Lakes
Source: PLoS One. 2021 May 28;16(5):e0232923. doi: 10.1371/journal.pone.0232923 (PMC8162641; doi:10.1371/journal.pone.0232923)

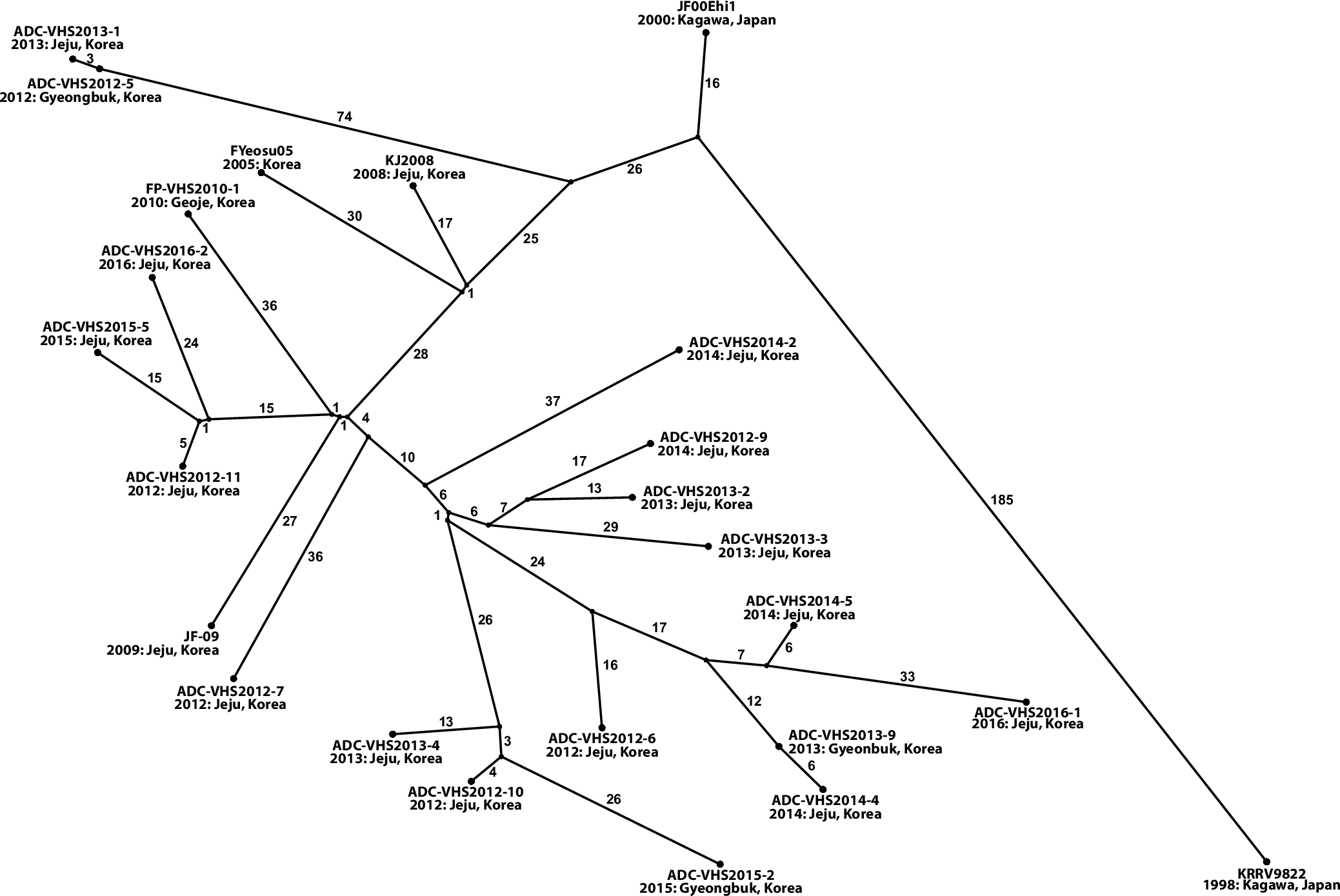

Supplement: S1 Fig — Circles sized according to haplotype frequency among isolates. Numbers inside parentheses designate NT differences between each node, unlabeled black circles = hypothesized haplotype steps. Year and location of isolation are below isolate names. (TIF) [file pone.0232923.s001.tif]

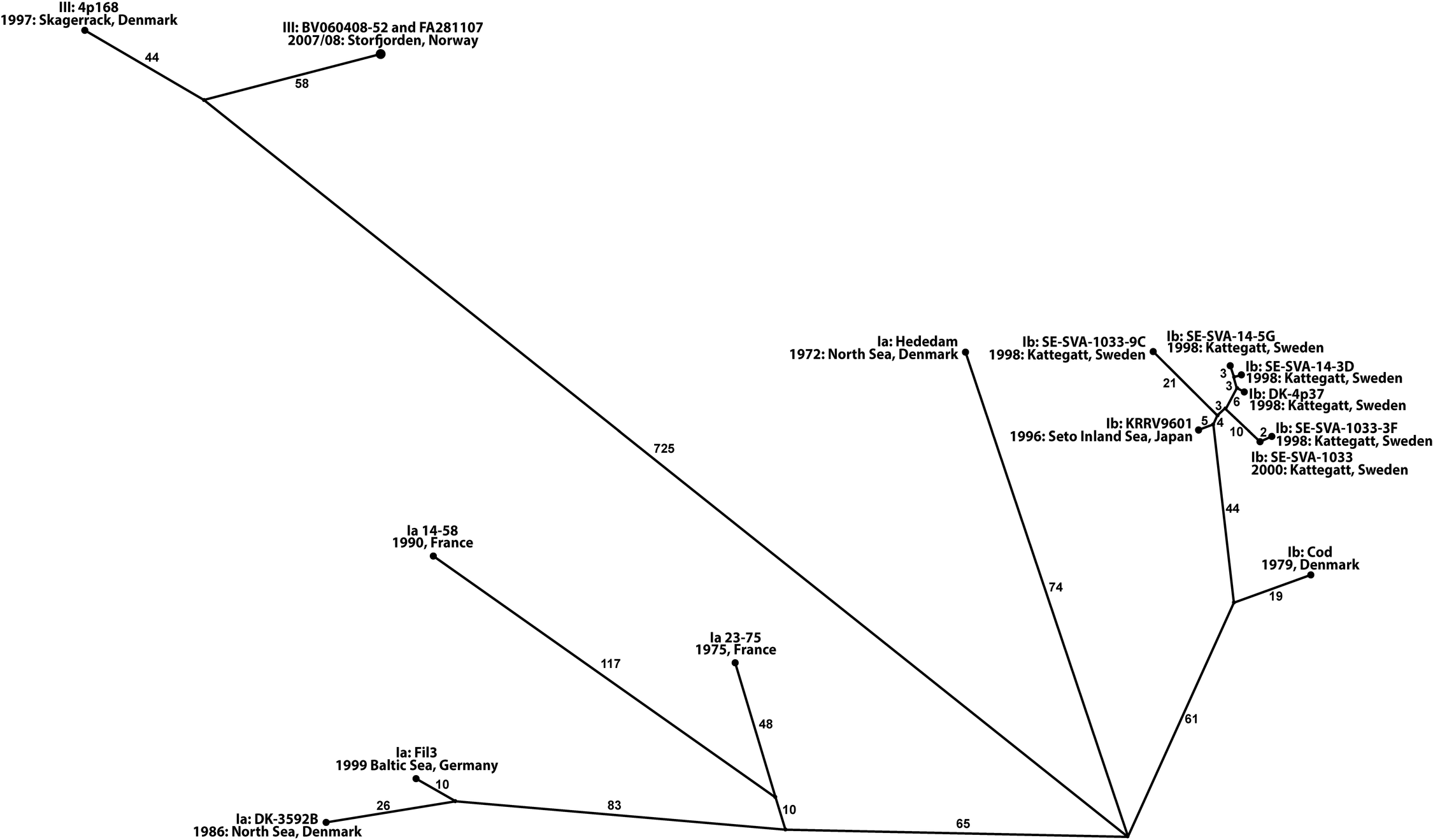

Supplement: S2 Fig — Circles sized according to haplotype frequency among isolates. Numbers inside parentheses designate NT differences between each node. Small, unlabeled black circles = hypothesized haplotype steps. Year and location of isolation are below isolate names. (TIF) [file pone.0232923.s002.tif]
