# Supplementary material for: Genomic and immunogenic changes of Piscine novirhabdovirus (Viral Hemorrhagic Septicemia Virus) over its evolutionary history in the Laurentian Great Lakes
Source: PLoS One. 2021 May 28;16(5):e0232923. doi: 10.1371/journal.pone.0232923 (PMC8162641; doi:10.1371/journal.pone.0232923)
Supplement: S1 Table — [25, 41, 44, 51, 55, 79, 97, 98, 108–118]. (DOCX) [file pone.0232923.s003.docx]

**S1 Table. VHSV isolates for whole genome analysis.**

| Isolate Name | Genogroup | Host Species | Location | Collection Year | GenBank | Citation |
| --- | --- | --- | --- | --- | --- | --- |
| DK-Hededam | I | North sea cod | North Sea, Denmark | 1972 | Z93412 | [108] |
| French strain 07-71 | Ia | Rainbow trout | Seine-Maritime, France | 1971 | AJ233396 | Yan et al. unpublished |
| DK-3592B | “ ” | “ ” | North Sea, Denmark | 1986 | KC778774 | [97] |
| De-Fil3 | “ ” | “ ” | Baltic Sea, Germany | 1999 | Y18263 | [109] |
| FR--23-75 | Ia | Brown trout | France | 1975 | FN665788 | [41] |
| FR14-58 | Ia | Rainbow trout | France | 1990 | AF143863 | [114] |
| Cod-Ulcus | Ib | North sea cod | Denmark | 1979 | Z93414 | [109] |
| SE-SVA-1033-3F | “ ” | Rainbow trout | Kattegatt, Sweden | 1998 | AB839748 | [110] |
| SE-SVA-1033-9C | “ ” | “ ” | “ ” | “ ” | AB839747 | “ ” |
| SE-SVA-14-3D | “ ” | “ ” | “ ” | “ ” | AB839745 | “ ” |
| SE-SVA-14-5G | “ ” | “ ” | “ ” | “ ” | AB839746 | “ ” |
| SE-SVA-1033 | “ ” | “ ” | “ ” | 2000 | FJ460591 | [111] |
| KRRV9601 | “ ” | Olive flounder | Seto Inland Sea, Japan | 1996 | AB672614 | [112] |
| DKp37 | “ ” | Blue whiting | North Sea, Denmark | 1997 | FJ460590 | [111] |
| DK-1p49 | II | Atlantic herring | Baltic Sea | 1996 | KM244767 | [113] |
| GH40 | III | Greenland halibut | Flemish Cap, Newfoundland | 1994 | KM244768 | [113] |
| 4p168 | “ ” | Atlantic herring | Skagerrack, Denmark | 1996 | AB672616 | [112] |
| FA281139 | “ ” | Rainbow trout | Storfjorden, Norway | 2007 | EU481506 | [115] |
| BV060408-52 | “ ” | “ ” | “ ” | 2008 | FJ362510 | “ ” |
| KRRV9822 | IVa | Olive flounder | Kagawa, Japan | 1998 | AB179621 | Byon et al. unpublished |
| JF00Ehi1 | “ ” | Japanese flounder | Ehime, Japan | 2000 | AB490792 | [112] |
| FYeosu05 | “ ” | “ ” | South Korea | 2005 | KF477302 | [116] |
| *Paralichthys olivaceus* rhabdovirus (VHSV) | “ ” | “ ” | China | “ ” | KC685626 | [117] |
| KJ2008 | “ ” | “ ” | Jeju, Korea | 2008 | JF792424.1 | [25] |
| JF-09 | “ ” | “ ” | “ ” | 2009 | KM926343 | [98] |
| FP-VHS2010-1 | “ ” | “ ” | Geoje, Korea | 2010 | KP334106 | [117, 118] |
| ADC-VHS2012-10 | “ ” | “ ” | Jeju, Korea | 2012 | KY979950 | “ ” |
| ADC-VHS2012-11 | “ ” | “ ” | “ ” | “ ” | KY979951 | “ ” |
| ADC-VHS2012-5 | “ ” | “ ” | Gyeongbuk, Korea | “ ” | KY979946 | “ ” |
| ADC-VHS2012-6 | “ ” | “ ” | Jeju, Korea | “ ” | KY979947 | “ ” |
| ADC-VHS2012-7 | “ ” | “ ” | “ ” | “ ” | KY979948 | “ ” |
| ADC-VHS2012-9 | “ ” | “ ” | “ ” | “ ” | KY979949 | “ ” |
| ADC-VHS2013-1 | “ ” | “ ” | “ ” | “ ” | KY979952 | “ ” |
| ADC-VHS2013-2 | “ ” | “ ” | “ ” | “ ” | KY979953 | “ ” |
| ADC-VHS2013-3 | IVa | Olive flounder | Jeju, Korea | 2013 | KY979954 | [79, 118] |
| ADC-VHS2013-4 | “ ” | “ ” | “ ” | “ ” | KY979955 | “ ” |
| ADC-VHS2013-9 | “ ” | “ ” | Gyeongbuk, Korea | “ ” | KY979956 | “ ” |
| ADC-VHS2014-2 | “ ” | “ ” | Jeju, Korea | 2014 | KY979957 | “ ” |
| ADC-VHS2014-4 | “ ” | “ ” | “ ” | “ ” | KY979958 | “ ” |
| ADC-VHS2014-5 | “ ” | “ ” | “ ” | “ ” | KY979959 | “ ” |
| ADC-VHS2015-2 | “ ” | “ ” | “ ” | 2015 | KY979960 | “ ” |
| ADC-VHS2015-5 | “ ” | “ ” | “ ” | “ ” | KY979961 | “ ” |
| ADC-VHS2016-1 | “ ” | “ ” | “ ” | 2016 | KY979962 | “ ” |
| ADC-VHS2016-2 | “ ” | “ ” | “ ” | “ ” | KY979963 | “ ” |
| C03MU* (=M103GL) | IVb | Muskellunge | L. St. Clair, USA | 2003 | GQ385941 | [55] |
| E06FD | “ ” | Freshwater drum | L. Erie, USA | 2006 | MK783014 | This study +[51] |
| E06WA | “ ” | Walleye | “ ” | “ ” | MK782987 | “ ” |
| E06WBa | “ ” | White bass | “ ” | “ ” | MK777861 | “ ” |
| E06YPa | “ ” | Yellow perch | “ ” | “ ” | MK782985 | “ ” |
| E06SB | “ ” | Smallmouth bass | “ ” | “ ” | MK782984 | “ ” |
| E06YPb | “ ” | Yellow perch | “ ” | “ ” | MK782983 | “ ” |
| E06YPc | “ ” | “ ” | “ ” | “ ” | MK782982 | “ ” |
| E06WBb | “ ” | White bass | “ ” | “ ” | MK783013 | “ ” |
| O06RG | “ ” | Round goby | L. Ontario, USA | 2006 | KY359357 | [44] |
| C06NP | “ ” | Northern pike | L. St. Clair, USA | 2006 | MK782990 | This study +[51] |
| C06GS | “ ” | Gizzard shad | “ ” | “ ” | MK777875 | “ ” |
| C06RB | “ ” | Rock bass | “ ” | “ ” | MK782990 | “ ” |
| C06SR | “ ” | Shorthead redhorse | “ ” | “ ” | “ ” | “ ” |
| C06YP | “ ” | Yellow perch | “ ” | “ ” | “ ” | “ ” |
| C06FD | “ ” | Freshwater drum | “ ” | “ ” | “ ” | “ ” |
| B07BG | “ ” | Bluegill | Budd L., MI, USA | 2007 | MK783006 | “ ” |
| B07PS | “ ” | Pumpkinseed | “ ” | “ ” | MK783008 | “ ” |
| E07CC | “ ” | Common carp | L. Erie, USA | “ ” | MK783005 | “ ” |
| E07YPa | “ ” | Yellow perch | “ ” | “ ” | MK782989 | “ ” |
| E07YPb | “ ” | “ ” | “ ” | “ ” | MK782988 | “ ” |
| M07SB | “ ” | Smallmouth bass | L. Michigan, USA | “ ” | MK783009 | “ ” |
| M08RB | “ ” | Rock bass | “ ” | 2008 | MK783010 | “ ” |
| E08ES | “ ” | Emerald shiner | L. Erie, USA | “ ” | MK783012 | “ ” |
| E08FDa | “ ” | Freshwater drum | “ ” | “ ” | MK782993 | “ ” |
| E08FDb | “ ” | “ ” | “ ” | “ ” | MK782992 | “ ” |
| M08AM | “ ” | Amphipod | L. Michigan, USA | 2008 | MK782990 | “ “ |
| C08Lea | “ ” | Leech | L. St. Clair, USA | 2008 | “ ” | “ “ |
| C08LEb | “ ” | “ ” | “ ” | “ ” | “ ” | “ ” |
| M08YP | “ ” | Yellow perch | L. Michigan, USA | “ ” | MK783007 | “ ” |
| C09MU | “ ” | Muskellunge | L. St. Clair, USA | 2009 | MK782990 | “ ” |
| M11YP | “ ” | Yellow perch | L. Michigan, USA | 2011 | MK782991 | “ ” |
| E12FD | “ ” | Freshwater drum | L. Erie, USA | 2012 | MK783004 | “ ” |
| O13GS | “ ” | Gizzard shad | L. Ontario, USA | 2013 | KY359355 | [44] |
| E14GS | “ ” | “ ” | L. Erie, USA | 2014 | KY359356 | “ ” |
| E15RG | “ ” | Round goby | “ ” | 2015 | MK783003 | This study +[51] |
| E16GSa | “ ” | Gizzard shad | “ ” | 2016 | MK783011 | “ ” |
| E16GSb | “ ” | “ ” | “ ” | “ ” | MK782997 | “ ” |
| E16GSc | “ ” | “ ” | “ ” | “ ” | MK782996 | “ ” |
| E16GSd | “ ” | “ ” | “ ” | “ ” | MK782994 | “ ” |
| E16GSe | “ ” | “ ” | “ ” | “ ” | MK782995 | “ ” |
| M16RGa | “ ” | Round goby | L. Michigan, USA | “ ” | MK783001 | “ ” |
| M16RGb | “ ” | “ ” | “ ” | “ ” | MK783000 | “ ” |
| CellC03 | “ ” | Muskellunge | L. St Clair, USA | 2003 | MK782981 | “ ” |
| Cell16a | “ ” | Gizzard shad | L. Erie, USA | 2016 | MK782998 | “ ” |
| Cell16b | “ ” | Largemouth bass | “ ” | “ ” | MK783002 | “ ” |
| Cell16c | “ ” | “ ” | “ ” | “ ” | MK782999 | “ ” |
