# Supplementary material for: Genomic and immunogenic changes of Piscine novirhabdovirus (Viral Hemorrhagic Septicemia Virus) over its evolutionary history in the Laurentian Great Lakes
Source: PLoS One. 2021 May 28;16(5):e0232923. doi: 10.1371/journal.pone.0232923 (PMC8162641; doi:10.1371/journal.pone.0232923)
Supplement: S2 Table — [21, 109, 119–126]. (DOCX) [file pone.0232923.s004.docx]

**S2 Table.** Additional *Rhabdovirus* sequences used in phylogenetic trees.

| Isolate Name | Host | Location | Year | GenBank | Sequence source |
| --- | --- | --- | --- | --- | --- |
| *Salmonid novirhabdovirus* (IHNV) | | | | | |
| X89213 | Rainbow trout | Oregon, USA | 1969 | X89213 | [109] |
| WRAC | Chinook salmon | Idaho, USA | 1994 | L40883 | [119] |
| “ ” | “ ” | “ ” | “ ” | NC_001652 | “ ” |
| 220-90 | Rainbow trout | Idaho, USA | 1990 | GQ413939 | [120] |
| HLJ-09 | “ ” | China | 2009 | JX649101 | [121] |
| Ch20101028 | Brook trout | “ ” | 2010 | KJ421216 | [122] |
| BjLL | Rainbow trout | “ ” | 2012 | MF509592 | [123] |
| *Snakehead novirhabdovirus* (SHRV) | | | | | |
| NC_000903 | Snakehead murrel | Thailand | 1988 | NC_000903 | [124] |
| AF147498 | “ ” | “ ” | “ ” | AF147498 | [21] |
| Hirame *novirhabdovirus* (HIRRV) | | | | | |
| CA 9703 | Japanese flounder | Japan | 1984 | NC_005093 | [125] |
| “ ” | “ ” | “ ” | “ ” | AF104985 | “ ” |
| 80113 | Stone flounder | China | 2008 | FJ376982 | [126] |
