# Supplementary material for: Genomic and immunogenic changes of Piscine novirhabdovirus (Viral Hemorrhagic Septicemia Virus) over its evolutionary history in the Laurentian Great Lakes
Source: PLoS One. 2021 May 28;16(5):e0232923. doi: 10.1371/journal.pone.0232923 (PMC8162641; doi:10.1371/journal.pone.0232923)
Supplement: S3 Table — * = Group, includes C06NP, C06RB, C06SR, C06YP, C06FD, E06WBc, M08AMa,b, C08LEa,b, and C09MU. (DOCX) [file pone.0232923.s005.docx]

**S3 Table.** Single nucleotide polymorphisms (SNPs) and nonsynonymous changes per individual isolates. *=Group, includes C06NP, C06RB, C06SR, C06YP, C06FD, E06WBc, M08AMa,b, C08LEa,b, and C09MU.

|  | ***N*-gene** | | ***P*-gene** | | ***M*-gene** | | ***G*-gene** | | ***Nv*-gene** | | ***L*-gene** | | **NCDS** | **Total** | | **Average** | |  |
| --- | --- | --- | --- | --- | --- | --- | --- | --- | --- | --- | --- | --- | --- | --- | --- | --- | --- | --- |
| **Isolate** | **NT** | **AA** | **NT** | **AA** | **NT** | **AA** | **NT** | **AA** | **NT** | **AA** | **NT** | **AA** | **NT** | **NT** | **AA** | **NT** | **AA** | **dN/dS** |
| E06FD | 1 | 0 | 0 | 0 | 0 | 0 | 0 | 0 | 0 | 0 | 3 | 0 | 0 | 4 | 0 | 1.6% | 0.0% | 0.000 |
| E06WA | 0 | 0 | 0 | 0 | 1 | 1 | 1 | 1 | 2 | 1 | 2 | 1 | 1 | 7 | 4 | 2.8% | 4.7% | 0.571 |
| E06WBb | 0 | 0 | 0 | 0 | 1 | 1 | 0 | 0 | 0 | 0 | 3 | 1 | 1 | 5 | 2 | 2.0% | 2.4% | 0.400 |
| E06YPa | 0 | 0 | 0 | 0 | 1 | 1 | 2 | 0 | 0 | 0 | 2 | 1 | 1 | 6 | 2 | 2.4% | 2.4 | 0.333 |
| E06SB | 1 | 0 | 0 | 0 | 1 | 1 | 1 | 1 | 0 | 0 | 2 | 1 | 1 | 6 | 3 | 2.4% | 3.5% | 0.500 |
| E06YPb | 0 | 0 | 0 | 0 | 1 | 1 | 0 | 0 | 0 | 0 | 2 | 1 | 0 | 3 | 2 | 1.2% | 2.4% | 0.667 |
| E06YPc | 0 | 0 | 0 | 0 | 1 | 1 | 1 | 1 | 0 | 0 | 2 | 1 | 1 | 5 | 3 | 2.0% | 3.5% | 0.600 |
| E06WBb | 0 | 0 | 1 | 0 | 1 | 1 | 3 | 3 | 0 | 0 | 3 | 1 | 1 | 9 | 5 | 3.6% | 5.9% | 0.556 |
| O06RG | 1 | 0 | 0 | 0 | 2 | 2 | 6 | 3 | 1 | 1 | 11 | 3 | 1 | 22 | 9 | 8.7% | 10.6% | 0.409 |
| C06NP* | 0 | 0 | 0 | 0 | 0 | 0 | 0 | 0 | 0 | 0 | 1 | 1 | 0 | 1 | 1 | 0.4% | 1.2% | 1.000 |
| C06GS | 1 | 0 | 0 | 0 | 2 | 1 | 1 | 1 | 2 | 0 | 3 | 1 | 5 | 14 | 3 | 5.5% | 3.5% | 0.214 |
| B07BG | 0 | 0 | 0 | 0 | 0 | 0 | 2 | 0 | 2 | 1 | 0 | 0 | 3 | 7 | 1 | 2.8% | 1.2% | 0.143 |
| B07PS | 1 | 0 | 0 | 0 | 0 | 0 | 1 | 0 | 0 | 0 | 3 | 2 | 1 | 6 | 2 | 2.4% | 2.4% | 0.333 |
| E07CC | 1 | 0 | 0 | 0 | 1 | 1 | 2 | 1 | 0 | 0 | 3 | 2 | 1 | 8 | 4 | 3.2% | 4.7% | 0.500 |
| E07YPa | 0 | 0 | 0 | 0 | 1 | 1 | 2 | 2 | 1 | 1 | 2 | 1 | 2 | 8 | 5 | 3.2 | 5.9% | 0.625 |
| E07YPb | 0 | 0 | 0 | 0 | 1 | 1 | 2 | 2 | 0 | 0 | 3 | 2 | 2 | 8 | 5 | 3.2 | 5.9 | 0.625 |
| M07SB | 1 | 0 | 0 | 0 | 1 | 1 | 3 | 3 | 0 | 0 | 3 | 1 | 0 | 8 | 5 | 3.2 | 5.9 | 0.625 |
| M08RB | 1 | 1 | 0 | 0 | 0 | 0 | 7 | 4 | 0 | 0 | 6 | 2 | 0 | 14 | 7 | 5.5% | 8.2% | 0.500 |
| E08ES | 2 | 2 | 2 | 2 | 1 | 1 | 0 | 0 | 0 | 0 | 8 | 3 | 1 | 14 | 8 | 5.5% | 9.4% | 0.571 |
| E08FDa | 0 | 0 | 0 | 0 | 1 | 1 | 0 | 0 | 0 | 0 | 3 | 1 | 1 | 5 | 2 | 2.0% | 2.4% | 0.400 |
| E08FDb | 0 | 0 | 0 | 0 | 1 | 1 | 1 | 0 | 0 | 0 | 4 | 1 | 1 | 7 | 2 | 2.8% | 2.4 | 0.286 |
| M08YP | 1 | 1 | 0 | 0 | 1 | 0 | 2 | 1 | 0 | 0 | 8 | 4 | 0 | 12 | 6 | 4.7% | 7.1% | 0.500 |
| M11YP | 0 | 0 | 1 | 1 | 1 | 1 | 0 | 0 | 2 | 0 | 3 | 2 | 2 | 9 | 4 | 3.6% | 4.7% | 0.444 |
| E12FD | 5 | 2 | 2 | 1 | 2 | 1 | 1 | 1 | 1 | 1 | 18 | 6 | 6 | 35 | 12 | 13.8% | 14.1% | 0.343 |
| O13GS | 4 | 1 | 2 | 0 | 2 | 1 | 8 | 3 | 0 | 0 | 9 | 2 | 4 | 29 | 7 | 11.5% | 8.2% | 0.241 |
| E14GS | 2 | 0 | 1 | 0 | 1 | 1 | 6 | 2 | 0 | 0 | 15 | 5 | 2 | 27 | 8 | 10.7% | 9.4% | 0.296 |
| E15RG | 4 | 2 | 2 | 0 | 1 | 0 | 8 | 4 | 1 | 0 | 9 | 3 | 4 | 29 | 9 | 11.5% | 10.6% | 0.310 |
| E16GSa | 3 | 1 | 3 | 0 | 1 | 1 | 6 | 6 | 2 | 1 | 16 | 5 | 6 | 37 | 14 | 14.6% | 16.5% | 0.378 |
| E16GSb | 3 | 1 | 3 | 0 | 1 | 1 | 7 | 7 | 2 | 1 | 16 | 5 | 6 | 38 | 15 | 15.0% | 17.6% | 0.395 |
| E16GSc | 3 | 2 | 3 | 0 | 1 | 1 | 3 | 3 | 2 | 2 | 4 | 1 | 3 | 19 | 9 | 7.5% | 10.6% | 0.474 |
| E16GSd | 3 | 1 | 3 | 0 | 0 | 0 | 3 | 3 | 2 | 2 | 16 | 5 | 5 | 32 | 11 | 12.6% | 12.9% | 0.344 |
| E16GSe | 4 | 2 | 3 | 0 | 0 | 0 | 5 | 5 | 2 | 1 | 16 | 5 | 6 | 36 | 13 | 14.2% | 15.3% | 0.361 |
| M16RGa | 2 | 1 | 1 | 0 | 4 | 1 | 1 | 1 | 1 | 0 | 4 | 1 | 1 | 14 | 4 | 5.5% | 4.7% | 0.286 |
| M16RGb | 3 | 1 | 4 | 0 | 3 | 1 | 1 | 1 | 1 | 0 | 6 | 0 | 0 | 18 | 3 | 7.1% | 3.5% | 0.167 |
| Cell16a | 4 | 1 | 3 | 0 | 1 | 1 | 6 | 6 | 2 | 1 | 16 | 5 | 6 | 38 | 14 | 15.0% | 16.5% | 0.368 |
| Cell16b | 4 | 1 | 3 | 0 | 1 | 1 | 6 | 6 | 2 | 1 | 12 | 4 | 6 | 34 | 13 | 13.4% | 15.3% | 0.382 |
| Cell16c | 4 | 1 | 3 | 0 | 1 | 1 | 6 | 6 | 2 | 1 | 13 | 4 | 6 | 35 | 13 | 13.8% | 15.3 | 0.382 |
| CellC03 | 0 | 0 | 0 | 0 | 1 | 1 | 3 | 1 | 0 | 0 | 0 | 0 | 0 | 4 | 2 | 1.6% | 2.4% | 0.500 |
| **AVERAGES** | 1.553 | 0.553 | 1.053 | 0.105 | 1.053 | 0.789 | 2.816 | 2.053 | 0.789 | 0.395 | 6.579 | 2.211 | 2.289 | 16.132 | 6.105 | 0.064 | 0.072 | 0.422 |
